# Interpretable representation learning for 3D multi-piece intracellular structures using point clouds

**DOI:** 10.1038/s41592-025-02729-9

**Published:** 2025-07-03

**Authors:** Ritvik Vasan, Alexandra J. Ferrante, Antoine Borensztejn, Christopher L. Frick, Philip Garrison, Nathalie Gaudreault, Saurabh S. Mogre, Fatwir S. Mohammed, Benjamin Morris, Guilherme G. Pires, Daniel Saelid, Susanne M. Rafelski, Julie A. Theriot, Matheus P. Viana

**Affiliations:** 1 Allen Institute for Cell Science, Seattle, WA USA; 2 Department of Biology and Howard Hughes Medical Institute, University of Washington, Seattle, WA USA; 3 Present Address: Altius Institute for Biomedical Sciences, Seattle, WA USA

**Keywords:** Cellular imaging, Organelles, Image processing, Machine learning, Data publication and archiving

## Abstract

A key challenge in understanding subcellular organization is quantifying interpretable measurements of intracellular structures with complex multi-piece morphologies in an objective, robust and generalizable manner. Here we introduce a morphology-appropriate representation learning framework that uses three-dimensional rotation-invariant autoencoders and point clouds. This framework is used to learn representations of complex shapes that are independent of orientation, compact and interpretable. We apply our framework to intracellular structures with punctate morphologies (for example, DNA replication foci) and polymorphic morphologies (for example, nucleoli). We explore the trade-offs in the performance of this framework compared to image-based autoencoders by performing multi-metric benchmarking across efficiency, generative capability and representation expressivity metrics. We find that the proposed framework, which embraces the underlying morphology of multi-piece structures, can facilitate the unsupervised discovery of subclusters for each structure. We show how this approach can also be applied to phenotypic profiling using a dataset of nucleolar images following drug perturbations.

## Abstract (web summary)

An image analysis framework makes use of rotation-invariant autoencoders and point clouds for morphology-appropriate representation learning to better understand the organization of complex subcellular structures.

## Main

A central goal of cell biology is to understand the spatial and dynamic organization of the components within the cell and how their interactions contribute to cell function. Enabled by advances in imaging methods, we are now at the dawn of the big data era for cellular imaging^1–4^, in which unprecedented amounts of rich image datasets can enable quantitative characterization of cellular organization and its connections with cellular phenotype.

The term cellular organization encompasses multiple aspects of a cell’s configuration that must be unpacked before further discussion. Here we focus on two of these aspects: spatial protein distributions and shape of multi-piece intracellular structures. For example, the spatial pattern of fluorescently labeled proliferating cell nuclear antigen (PCNA), representing the punctate morphology of DNA replication foci, changes throughout the cell cycle, making it difficult to quantify due to its dynamic and complex nature. These types of spatial distributions are usually analyzed via the texture patterns they represent, for example, computing Haralick texture features^5^. However, the biological meaning of some of these features, such as the ‘second angular moment of texture’, is difficult to understand. Therefore, with spatial protein distributions, we face the challenge of developing a robust and generalizable analysis workflow that facilitates biological interpretation.

On the other hand, major organelles or subcellular structures can often be analyzed by segmentation, which separates the foreground signal from the background. Intracellular structures composed of a single segmented piece, such as the cell itself or the nucleus, can then be studied via a range of features including, among other methods, shape decomposition using spherical harmonic expansion^1,6,7^. This approach is, however, mainly used for cell and nuclear shapes because it is limited to continuous shapes, and does not easily apply to complex, multi-piece structures like the Golgi apparatus, which has a discontinuous shape. In fact, most intracellular structures exhibit a polymorphic morphology consisting of multiple pieces, which presents another challenge for interpretable image analysis pipelines. While each individual piece could be segmented and measured, the entirety of the multi-piece structure cannot be easily represented as a whole. Therefore, we face two key challenges: the need for interpretable methods to analyze spatial protein distributions, and the difficulty in representing complex, multi-piece intracellular structures.

To overcome these two challenges, we demonstrate the use of three-dimensional (3D) point clouds to encode biological data in microscopy images, combined with an unsupervised ‘representation learning’ framework for single-cell feature extraction. Representation learning is a field of machine learning that has become an increasingly popular way to extract meaningful features directly from raw data without the need for hand-engineered features^8,9^. These features are in the form of latent variables learned by neural networks during training, which we refer to as ‘representations’.

An important aspect of the proposed learning framework is that it is generative, meaning we can transform learned representations back into the original point clouds and vice versa, resulting in highly interpretable features and addressing the first challenge described. A key contribution of this work is the use of point clouds to incorporate intensity information present in large 3D images representing spatial protein distribution in a segmentation-free manner. Furthermore, to address the challenge of analyzing multi-piece intracellular structures, we adapted the point cloud-based approach to handle segmented multi-piece shapes. This is achieved using the concept of signed distance field (SDF), allowing us to generalize our framework to more complex intracellular structures.

The representations learned by neural networks normally depend on the orientation of an object in the image. Even though the orientation of the cells is important in many contexts, such as when cells are subject to shear stress, during development or directed migration, it may not have biological relevance in other contexts. For example, the orientation of a cell within a monolayer colony grown on a substrate may merely reflect the orientation of that colony relative to the microscope stage and not anything biological. Therefore, it would be desirable to design analysis workflows where the image orientation can be factored out of the learned representations if appropriate. We achieved this by leveraging the notion of ‘3D rotation invariance’ to extract features that do not depend on an object’s orientation. The incorporation of geometric information in the form of the object orientation into the representation learning process is an example of ‘geometric deep learning’^10^. By using point clouds as a unifying way of encoding image data, we are able to overcome the challenges described above and take advantage of previous implementations for rotation-invariant feature learning^11–13^ while extending their applications to quantitative cell biology.

Here, we first develop a rotation-invariant representation learning framework that uses point clouds to encode relevant information about the underlying biological data. We then use a synthetic dataset of punctate structures to confirm that rotation-invariant representations are not sensitive to data orientation and are more compact when learned from data encoded as point clouds compared to when they are learned from microscopy images directly. We show how 3D rotation-invariant features learned from point clouds can be used to recover unique morphological changes of DNA replication foci across the cell cycle without supervision. We also explore the localization patterns of several other punctate structures and discover new patterns of intracellular organization. Next, by adapting our framework to handle more complex multi-piece structures, we systematically characterize sources of shape variation of other major intracellular structures including nucleoli, Golgi and lysosomes. Finally, we demonstrate how the learned representations based on this framework can be used for detecting morphological alterations in a nucleolar drug perturbation dataset, and for visualizing the average phenotype for each drug to aid in interpretability of the phenotype.

## Results

### A framework for morphology-appropriate representation learning

**Fig. 1:**
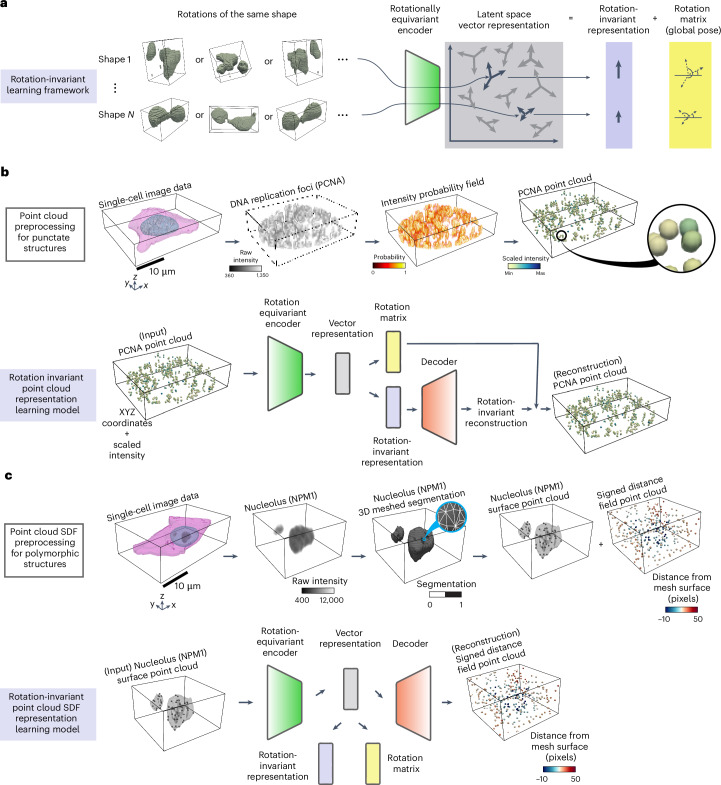
Application-appropriate representation learning framework for complex intracellular structure morphologies. **a**, 3D rotation-invariant representation learning framework using rotation-equivariant encoders. Rotations of the same shape are projected into vector representations using a 3D rotation-equivariant encoder. The norm of the vector representation is used to compute the rotation-invariant representation. The orientation of the vector representation is used to compute the rotation matrix. **b**, Point cloud learning framework for punctate structures like DNA replication foci. Top row, point cloud preprocessing for punctate structures. Shown are single-cell segmentations for the nucleus and cell membrane, and raw intensities for DNA replication foci (via PCNA). Four-dimensional (4D; *XYZ* + intensity) point clouds are sampled from the intensity images by converting intensities to probabilities. The intensity coordinate is scaled to ensure that the range of intensity values is like the range of *XYZ* coordinate values. Bottom row, rotation-invariant point cloud representation learning model. The 4D point cloud is used as input to the rotation-equivariant encoder. The decoder reconstructs the rotation-invariant representation to obtain a rotation-invariant reconstruction. The reconstruction is reoriented using the learned rotation matrix. **c**, Point cloud learning framework for polymorphic structures like the GC of nucleoli (via nucleophosmin). Top row, point cloud SDF preprocessing for polymorphic structures. Shown are single-cell segmentations for the nucleus, the cell membrane and nucleoli (GC). Nucleoli segmentation from single-cell data is used to generate a 3D mesh. A surface point cloud is sampled from the nucleolar mesh. Another point cloud is sampled from the 3D bounding box volume and its points are assigned local SDF values relative to the surface of the nucleolar mesh. Bottom row, rotation-invariant point cloud SDF representation learning model. The surface point cloud is used as input to the rotation-equivariant encoder. The decoder reconstructs the vector representations to obtain the SDF point cloud. The rotation-invariant representation and the rotation matrix are computed from the vector representation.

The 3D rotation-invariant representation learning framework has two main components. The first component addresses a critical issue in biological image analysis that is capturing consistent structural information regardless of an object’s orientation in the image. We used a specialized neural network encoder that can ‘understand’ biological shapes consistently, even when they are rotated in 3D space. This neural network learns representations in a vector form. This is done in a way that multiple rotations of the same object are mapped into distinct rotations of the same vector^11^ (Fig. 1a and ‘Models’ in Methods).

The second component consists of encoding the raw single-cell image data into a point cloud that is then fed to the neural network for representation learning. This encoding process is done in a morphology-appropriate manner, and is thus slightly different for punctate structures, such as DNA replication foci, versus polymorphic intracellular structures, such as nucleoli. The biological meaning of shape differs between these two types of morphologies; we focus on encoding only the relative location of individual pieces in punctate structures (Fig. 1b and ‘Punctate structures’ in Methods), while both the relative location and the shape of individual pieces are considered important for polymorphic structures (Fig. 1c and ‘Polymorphic structure datasets’ in Methods).

We use these morphology-appropriate encodings as the input to our rotation-invariant representation learning framework designed as an ‘autoencoder’^14^: First, the 3D rotation-equivariant ‘encoder network’ compresses the generated point clouds into vector latent representations. Next, the latent representations are used by a ‘decoder network’ to reconstruct the input data. In the case of punctate structures, the decoder network reconstructs the input point cloud using a combination of the learned rotation-invariant representations and rotation matrices to reorient the reconstructed shape into the correct input orientation (Fig. 1b). In the case of polymorphic structures, the decoder network reconstructs the SDF point cloud (Fig. 1c and ‘Polymorphic structure datasets’ in Methods) from the vector latent representations, which are converted into rotation-invariant representations after training by taking their norms (‘Models’ in Methods).

To evaluate the utility of the 3D point cloud encoding, we performed benchmarking against traditional methods using neural network models trained on 3D images directly. We trained classical (rotation dependent) and rotation-invariant versions of both image-based and point cloud-based models to evaluate the impact of adding the geometric constraint of rotation invariance. We expected point cloud-based models to outperform image-based models for two reasons. First, point clouds are a less redundant way of encoding sparse multi-piece intracellular structures compared to image-based models. This is because a sparse intracellular structure occupies only a few voxels in 3D space (for example, ~8% of voxels in a 3D image of DNA replication foci correspond to the relevant signal of PCNA in late S–G2 cell-cycle stages), and consequently, most of the 3D space contains empty and redundant information. Sampling point clouds from the region occupied by the structure can help remove this redundancy. Second, image-based autoencoders often generate blurry reconstructions that can be particularly problematic for small objects^15,16^. More details about all models used herein can be found in ‘Models’ in Methods.

**Extended Data Fig. 1:**
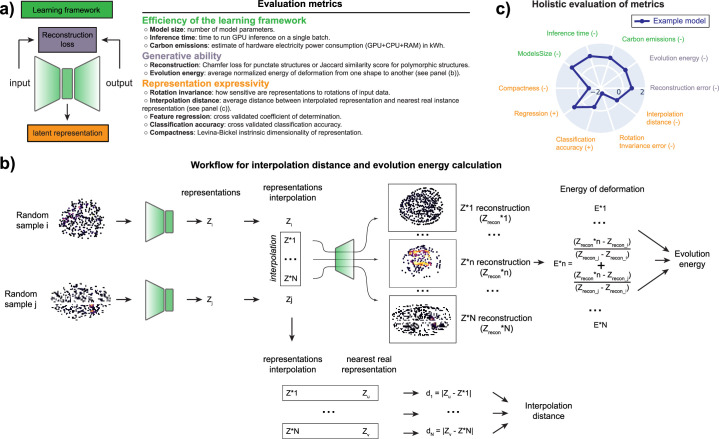
Evaluation metrics for representation learning. **a)** Overview of different evaluation metrics for quantifying the utility of each representation learning framework. Efficiency metrics include model size, inference time, and carbon emissions. Generative ability metrics include reconstruction error and evolution energy. Representation expressivity metrics include rotation invariance error, interpolation distance, feature regression, classification accuracy, and compactness. **b)** Workflow for interpolation distance and evolution energy calculation. Two samples are drawn from the population randomly, and a linear interpolation is performed on the representations of the two samples. The Euclidean distance between an interpolation and the nearest real representation is the interpolation distance. The interpolation distance is averaged across many interpolations to compute the average interpolation distance. Each interpolation is reconstructed using the decoder to obtain a reconstruction. The sum of the reconstruction error between the interpolated reconstruction and the reconstructions of the initial and final shapes normalized by the reconstruction error between the initial and final shape is the energy of deformation^7^. The energy of deformation is averaged across many interpolations to compute the evolution energy. Both evolution energy and average interpolation distance are averaged across many random pairs of samples from the test set. **c)** Holistic evaluation of metrics. Metrics are z-scored across models per metric. Z-scored metrics are visualized using a polar plot. We flip the sign for metrics where lower is better. Therefore, for all metrics, larger/outer values in the polar plot are better.

We used a multi-metric approach to evaluate our models and the representations learned by them. Our goal is to increase the transparency of the reasons for the performance of these models, and to explore trade-offs. Importantly, we hope to identify models that are quantitatively useful across a broad set of tasks to make gaining biological insight from the learned representations more likely, not necessarily the model that is best for any one metric. The models were evaluated with respect to their efficiency, generative capabilities and representation expressivity as detailed in Extended Data Fig. 1 (Supplementary Note 1). Considering all these metrics together, we quantified the holistic utility of each model and the advantages and disadvantages of using each approach.

### Synthetic data evaluation reveals holistic representations

**Fig. 2:**
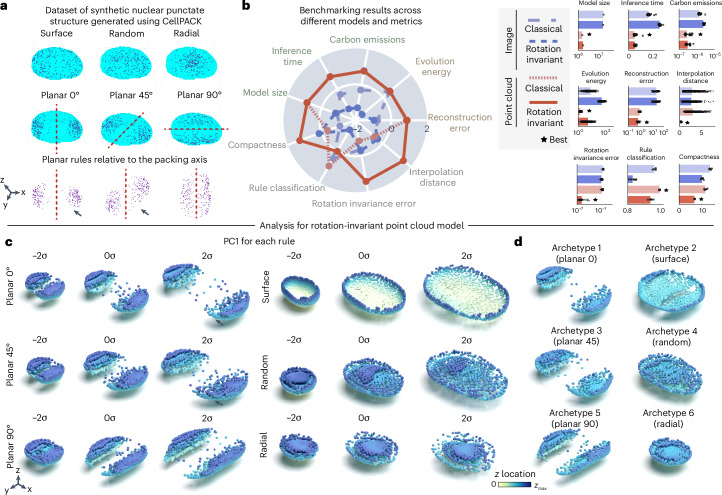
3D rotation-invariant point cloud models are efficient, produce low rotation invariance errors and generate good reconstructions in a synthetic dataset of punctate structures. **a**, Dataset of synthetic punctate structures generated using cellPACK. A 3D nuclear shape is packed with six different rules: planar 0, planar 45, planar 90, radial, random and surface. The surface rule packs spheres close to the nuclear boundary. The random rule packs spheres randomly across the 3D nuclear volume. The radial rule packs spheres close to the centroid. The planar rules pack spheres with a gradient away from a plane indicated in red. Each rule is used to pack 254 different nuclear shapes. The black arrows for planar 0 versus planar 45 highlight the symmetric versus asymmetric nature of these two packings in nuclei with high aspect ratios. **b**, Benchmarking unsupervised representations across different models and metrics. Left, polar plot showing the performance of classical and rotation-invariant image and point cloud models across efficiency metrics (model size (*n* = 1), inference time (*n* = 40) and emissions (*n* = 40)), generative metrics (reconstruction (*n* = 234) and evolution energy (*n* = 1,053)) and representation expressivity metrics (compactness (*n* = 5), classification of rules (*n* = 5), rotation invariance error (*n* = 936) and average interpolate distance (*n* = 1,053)). Metrics are *z*-scored and scaled such that larger is better. Right, bar plots showing raw metric values across models for each metric. Error bars are the s.d. The best model for each metric is indicated. **c**, PC1 for each rule using the rotation-invariant point cloud model trained with jitter augmentations. PCA is fit to representations of each rule separately. Shown are normalized PCs (s.d./*σ*) sampled at three map points (−2*σ* to 2*σ* in steps of *σ*). Black arrows for planar 0 versus planar 45 indicate the symmetric versus asymmetric reconstructions for these two packings at 2*σ*. **d**, Six archetypes computed from the rotation-invariant point cloud representations. Each archetype corresponds to one of the six rules. All reconstructions shown are cut at the midplane. Color associated with each point is the distance from the midplane in *Z*. Source data

We started by evaluating the effectiveness of 3D rotation invariance and the choice of using point clouds to encode punctate structures using synthetic data. We used cellPACK to create a synthetic dataset of punctate structures with known rules of organization. cellPACK generates 3D models of complex biological environments using novel packing algorithms^17^. To create the synthetic dataset, we used six spatial rules for packing spheres in real 3D nuclear shapes based on gradient algorithms (‘cellPACK synthetic single-cell dataset’ in Methods and Fig. 2a). Importantly, the stochastic nature of the packing algorithm generates heterogeneity in the distribution of spheres across the simulated nuclei that makes the recovery of rules via unsupervised learning difficult.

Since 3D rotation is an important variable associated with the planar rules, we expected 3D rotation-invariant models to give us the most compact representations by factoring out this variable. We additionally hypothesized that point cloud models (Fig. 1a) would provide better representations than image models because they better describe the punctate nature of the synthetic data represented by the centroid of the packed spheres (Fig. 2a). To test this hypothesis, we trained two classical and two 3D rotation-invariant models using images and point clouds as input data, respectively, resulting in four models for evaluation (‘Punctate structures’ and ‘Models’ in Methods and Supplementary Notes 2 and 3).

We found that point clouds displayed superior performance across all efficiency metrics (model size, inference time and emissions in Fig. 2b). In addition, point clouds also produced better reconstructions (‘reconstruction error’) and had low evolution energy scores, meaning that the interpolations between two shapes are smooth. We also confirmed that the implementations of the rotation-invariant models were indeed generating representations that were not sensitive to the orientation of the input data (Supplementary Fig. 1). Specifically, we evaluated that the model reconstructions given different orientations of the same input shapes were indistinguishable (Supplementary Note 4).

We found that rotation-invariant representations from point clouds were more compact using the Levina–Bickel intrinsic dimensionality metric (‘compactness’)^18^ and had much lower rotation invariance errors compared to their image-based counterpart (Fig. 2b). All four models were able to reconstruct the unique morphologies associated with each packing rule (Supplementary Fig. 2). However, we found that representations from both rotation-invariant models were slightly worse than their classical counterparts at classifying the six rules (‘rule classification’ in Fig. 2b). This was an expected outcome because rotation is an important distinguishing feature of the planar rules and rotation-invariant representations are insensitive to this feature. Overall, the 3D rotation-invariant point cloud model was an efficient generative model that learned compact and orientation-independent representations for synthetic punctate structures.

Having established the holistic utility of the rotation-invariant point cloud model on synthetic data, we next performed principal component analysis (PCA; Supplementary Note 5.1) on the learned representations using this model to interpret their meaning. We performed PCA on representations for each rule to assess their internal variability. By applying jitter augmentations (Supplementary Fig. 2g; see jitter details in Methods) during model training, we observed slightly improved reconstruction quality, especially for radial and planar rules (compare the reconstructions in Supplementary Fig. 2g to the reconstructions in Supplementary Fig. 2f). Consequently, we conducted subsequent PCA analyses using the jitter-augmented model. By visualizing the first principal component (PC1) of the reconstructions for each rule via a latent walk, we found that PC1 recovers how nuclear size affects each rule’s packing (Fig. 2c). Notably, the rotation-invariant reconstructions for all planar rules are aligned in the same direction, allowing us, for example, to focus on the subtle differences in spatial distribution between 0 and 45 degrees of orientation (Supplementary Note 4).

Next, we performed an archetype analysis^19^ to find extreme points in the representations of the synthetic dataset (Supplementary Note 5.2). Contrary to the PCA analysis, the archetype analysis was performed on the representations of all samples in the synthetic dataset regardless of its packing rule. Archetypes are determined so that observations can be approximated by convex combinations of the archetypes. By setting the number of archetypes to six, we found each archetype represented one of the six rules used in cellPACK to generate the synthetic dataset. These results show that the obtained point cloud rotation-invariant representations can enable unsupervised rule discovery for a synthetic dataset of punctate structures.

### Representations recover cell-cycle patterns of DNA replication foci

**Fig. 3:**
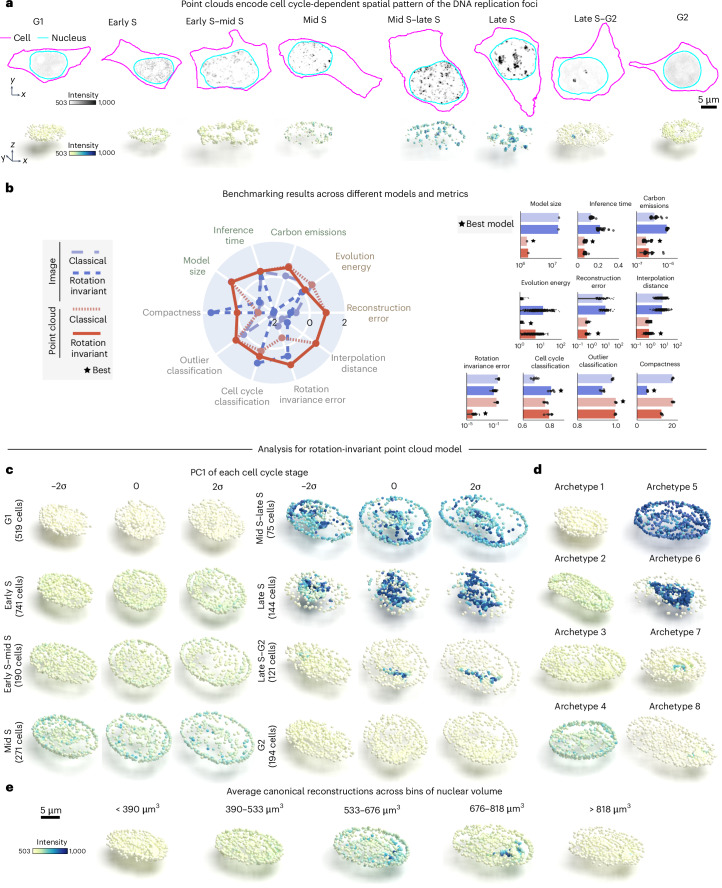
Rotation-invariant point cloud representations recover the cell-cycle-dependent spatial pattern of DNA replication foci. **a**, Dataset of DNA replication foci in hIPS cells expressing monomeric enhanced green fluorescent protein (mEGFP)-tagged PCNA. DNA replication foci have a stereotypical cell-cycle-dependent localization pattern. Shown are examples of image and sampled point cloud center slices with adjusted contrast for eight expert-annotated cell-cycle stages. **b**, Benchmarking unsupervised representations across different models and metrics. Left, polar plot showing performance of classical and rotation-invariant image and point cloud models across efficiency metrics (model size (*n* = 1), inference time (*n* = 40) and emissions (*n* = 40)), generative metrics (reconstruction (*n* = 122) and evolution energy (*n* = 180)) and representation expressivity metrics (compactness (*n* = 5), classification of cell cycle via top-2 classification accuracy (*n* = 5), rotation invariance error (*n* = 488) and average interpolate distance (*n* = 180)). Metrics are *z*-scored and scaled such that larger is better. Right, bar plots showing raw metric values across models for each metric. Error bars are standard deviations. The best model for each metric is indicated. **c**, Eight archetypes identified using rotation-invariant point cloud representations. Each archetype corresponds to one of the eight expert-annotated cell-cycle stages. **d**, PC1 for each cell-cycle stage using rotation-invariant point cloud model. PCA is fit to representations of each cell-cycle stage separately. Shown are normalized PCs (s.d./σ) sampled at three map points (−2σ to 2σ in steps of σ). **e**, Average canonical reconstructions across five bins of nuclear volume (Supplementary Note 5.3). All reconstructions shown are center slices. Source data

**Extended Data Fig. 2:**
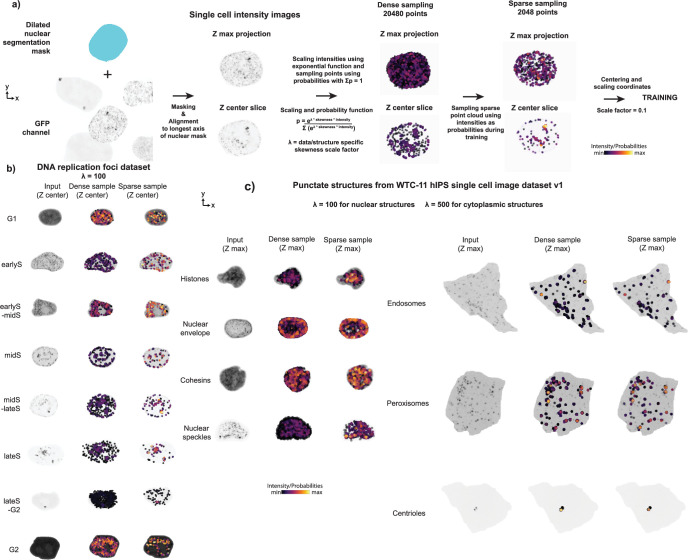
3D image preprocessing into application appropriate inputs for punctate structures. Workflow for generating 4D point clouds from 3D intensity images. **a)** single-cell intensity images are obtained by masking via a dilated nuclear mask (for nuclear structures), followed by alignment to the longest axis of the nuclear mask. Intensities were then scaled using an exponential function and then converted to probabilities. These probabilities were then used to sample a dense 4D point cloud with 20480 points and XYZ + intensity coordinates. During training, a sparse point cloud with 2048 points was sampled from this dense point cloud using the intensities as probabilities. The intensity coordinate was scaled using a scale factor of 0.1 to ensure that intensity values were in the same range as XYZ coordinate values. **b)** Examples of dense sample and sparse sample for each cell cycle stage for PCNA dataset. Shown are center-slice of raw intensity image, center-slice of raw intensity image overlaid with dense sample, and center-slice of raw intensity image overlaid with sparse training sample. **c)** Examples of dense sample and sparse sample for each punctate structure from the WTC-11 hiPSC Single-Cell Image Dataset v1. Structures include histones, nuclear envelope, cohesins, nuclear speckles, endosomes, peroxisomes, and centrioles.

After establishing its applicability to synthetic data, we tested the representation learning framework on a real single-cell image dataset of punctate structures for biological discovery and hypothesis generation. The dataset contains single-cell images of DNA replication foci in human induced pluripotent stem (hiPS) cells expressing fluorescently tagged PCNA (*N* = 2,420; ‘DNA replication foci dataset’ in Methods). DNA replication foci are punctate and display a continuous change in their overall localization pattern and intensity throughout cell cycle^20^ (Fig. 3a). Due to tagged PCNA fluorescence intensity being an important source of variation for DNA replication foci patterns, we adapted the point cloud sampling strategy so that the raw image intensity is treated as a fourth coordinate, in addition to the *XYZ* spatial coordinates (Extended Data Fig. 2a,b and ‘DNA replication foci dataset’ in Methods). This additional coordinate ensures that intensity information is captured in the learned representations (‘Point cloud models’ in Methods).

To test whether the representations learned with these data capture biologically relevant features about DNA replication foci localization, we manually classified each single-cell image in this dataset into one of eight cell-cycle stages based on the spatial pattern of PCNA (‘DNA replication foci dataset’ in Methods). We also manually labeled cells as outliers if they were dead, dying or did not express PCNA. Next, we used the representations learned by each of the four models to benchmark their performance on various tasks, including the application-appropriate task of classifying cell-cycle stages and detecting outliers from the DNA replication foci dataset.

We found that point cloud models were more efficient but, in this case, not as compact as the rotation-invariant image model (Fig. 3b). Point cloud models also provided better overall reconstructions compared to image models (compare reconstructions in Supplementary Fig. 3f–h to Supplementary Fig. 3b,c). Despite the poor reconstruction of both image models (Supplementary Fig. 3b,c), we found that the rotation-invariant image model was the best at classifying cell-cycle stages (‘Cell-cycle classification’ in Fig. 3b; 81% accuracy versus 80% accuracy for the best point cloud model). This result demonstrates the limits of evaluating models using a single metric alone. We confirmed that poor reconstructions of image models were not due to dataset size or image normalization issues using an alternative approach (Supplementary Fig. 3d and ‘Masked autoencoders using vision transformers’ of Methods). We also found that point cloud models had slightly better performance detecting outliers compared to image-based models (‘Outlier classification’ in Fig. 3b; ~100% accuracy versus 98% accuracy for the best image model). Finally, we found that the rotation-invariant point cloud model had lower rotation invariance error scores compared to its image counterpart. Overall, the results elucidate the challenge of reconstructing sparse intracellular structures using classical image autoencoders^15^, and highlight the good performance of the rotation-invariant point cloud representations across many metrics evaluated for the DNA replication foci dataset.

To interpret the representations learned by the rotation-invariant point cloud model for each cell-cycle stage, we performed PCA on the dataset, stratifying the analysis by manual cell-cycle stage annotations and fitting the PCA separately for each stage. As before, the PCA analysis is followed by a latent walk and data reconstruction that can be visually evaluated. A latent walk along PC1 for each cell-cycle stage revealed some overlap in the morphology and intensity of DNA replication foci between neighboring cell-cycle stages (early S *σ* = 2 and early S-mid S *σ* = −2, for example). This highlights the inherent uncertainty that is present in the task of manual annotation of a continuous process like cell cycle into discrete classes.

We found that an archetype analysis with eight archetypes on the representations of all cell-cycle stages was able to recover expected cell-cycle patterns of DNA replication foci (order of archetypes in Fig. 3d resembles examples in Fig. 3c for *σ* = 0). The archetypes capture three main sources of variation in the dataset as expected. The first is overall nuclear shape, which is mainly represented by archetypes displaying different nuclear sizes and elongations. In addition to nuclear shape, the intensity and localization of DNA replication foci are different between archetypes. These two sources of variation seemed consistent with what we observed in real PCNA images. Lastly, the spatial pattern of PCNA changes from a dim signal uniformly distributed in the nucleus at G1 to compact, well-localized bright spots in late S.

Next, we investigated whether the learned representations could capture known changes in the spatial patterns of DNA replication foci across the cell cycle in an unsupervised manner. Instead of using expert-generated labels, we derived pseudo labels based on nuclear volume. This was done by dividing the nuclear volume into five bins, each corresponding to a different portion of the cell cycle (Supplementary Note 5.3). For each bin, we computed the average representation of the DNA replication foci patterns by averaging the rotation-invariant point cloud representations of all cells in that bin (Fig. 3e). We visualized the average representations across the bins (Fig. 3e), and we observed that the size of the DNA replication foci point cloud increased with increasing nuclear size. We also observed a transition in the DNA replication foci pattern from a uniformly distributed dim set of puncta into a coalesced set of bright dots. This is reminiscent of the transition from G1 to late S. This pattern was then followed by signal sparsification into uniform dim punctate structures again, which is indicative of the transition from late S to G2. However, we observed that some subtle patterns from cell-cycle stages with small numbers like mid S–late S (*N* = 75) and late S (*N* = 144) were missing. Overall, these results demonstrate that the learned point cloud rotation-invariant representations can recover the overall biological behavior of DNA replication foci of well-represented cell-cycle stages in an unsupervised manner.

### Interpreting spatial patterns of other punctate structures

**Fig. 4:**
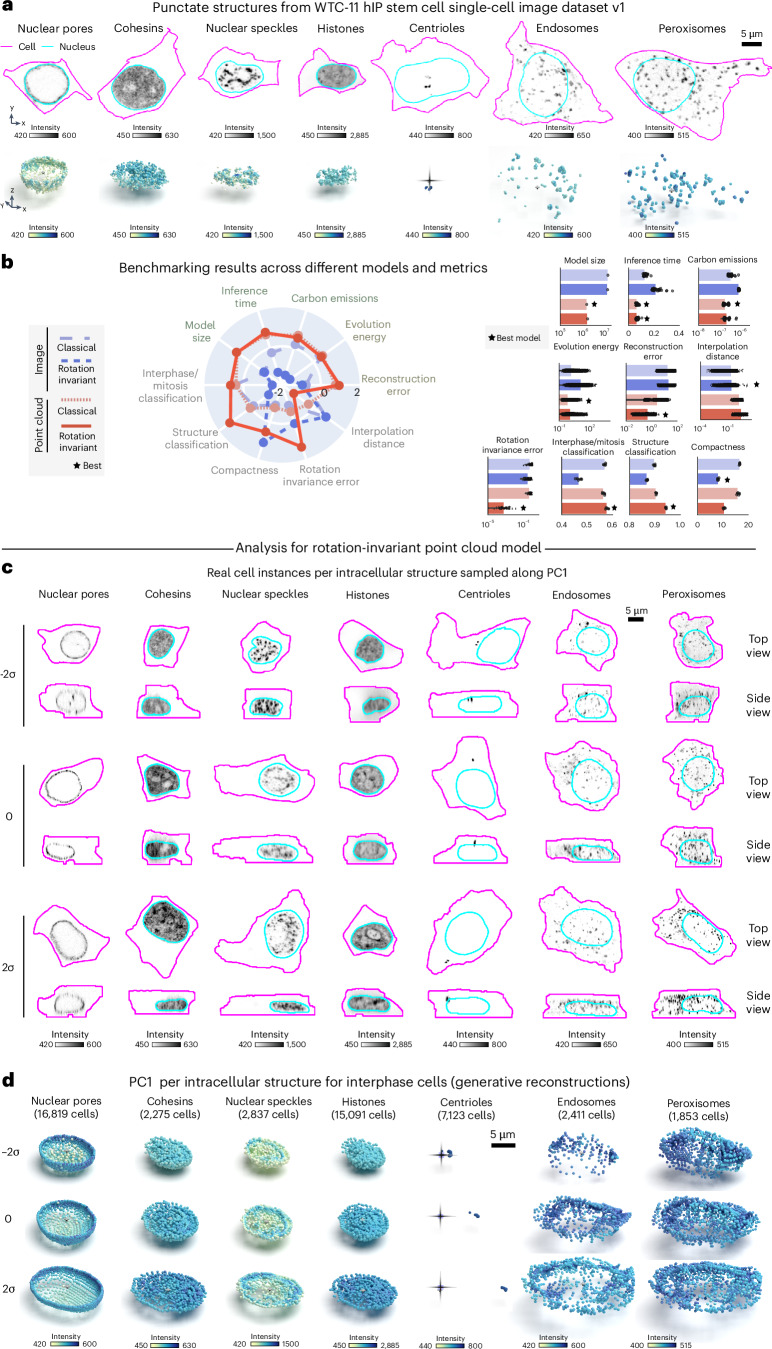
Representation learning framework reveals interpretable spatial patterns for other punctate structures from the WTC-11 hiPS cell single-cell image dataset v1. **a**, Dataset of punctate structures in hiPS cells from the WTC-11 hiPS cell single-cell image dataset v1 including nuclear pores, nuclear speckles, cohesins, histones, centrioles, peroxisomes and endosomes^1^. Shown are examples of images and sampled point cloud center slices of the mEGFP-tagged protein. **b**, Benchmarking unsupervised representations across classical and rotation-invariant image and point cloud models across efficiency metrics (model size (*n* = 1), inference time (n = 40) and emissions (*n* = 40)), generative metrics (reconstruction (*n* = 7,620) and evolution energy (*n* = 180)) and representation expressivity metrics (compactness (*n* = 5), classification (*n* = 5), rotation invariance error (*n* = 16,004) and average interpolate distance (*n* = 180)). Classification tasks included classifying seven different structures, and six different interphase/mitotic stages (Supplementary Note 1.2). Left, polar plot showing the performance across models where metrics are *z*-scored and scaled such that larger is better. Right, bar plots showing raw metric values across models for each metric. Error bars are the s.d. The best model for each metric is indicated. **c**, Real examples for each map point of PC1 computed using PCA fit to representations of each structure separately using the rotation-invariant point cloud model. Only cells in interphase were included. Shown are *XY* and *XZ* views. The structure channel is shown as center slices across the nuclear centroid for nuclear pores, cohesins and histones, or as maximum projections for nuclear speckles, centrioles, endosomes and peroxisomes. **d**, Latent walk for PC1. Shown are normalized PCs (s.d./σ) sampled at three map points (−2σ to 2σ in steps of σ). Reconstructions shown are cut at the midplane. Membrane centroids are marked for centrioles. Only cells in interphase were considered for this analysis. Centriole reconstructions were rotated to be aligned to the *x* axis. Source data

To assess whether our approach would generalize to other intracellular structures with punctate morphology, we analyzed a larger dataset of punctate structures from the WTC-11 hiPS cell single-cell image dataset v1 (Methods). This dataset comprises centrioles (*N* = 7,575), peroxisomes (*N* = 1,997), endosomes (*N* = 2,601), nuclear pores (*N* = 17,703), nuclear speckles (*N* = 2,980), cohesins (*N* = 2,380) and histones (*N* = 15,875). Examples of these structures are shown in Fig. 4a. Once again, we trained classical and rotation-invariant image-based and point cloud-based models on this larger dataset (Extended Data Fig. 2c; ‘Punctate structures’ in Methods). In addition to the usual set of evaluation metrics, we tested the applicability of the learned representations for two classification tasks. The first task focused on identifying the specific intracellular structures from the seven options available in the dataset. The second task involved classifying cell-cycle stages (interphase or mitosis) based on the annotations provided within the dataset (Supplementary Note 1.2).

Overall, we found that classical image-based models provide better reconstructions when trained with the combination of these seven different punctate structures relative to what we observed for the same models trained on the DNA replication foci dataset alone (Supplementary Fig. 4a,b). Despite this improvement in reconstruction, we noticed that the classical image-based model poorly reconstructs some of these structures including centrioles, peroxisomes and endosomes (see blurry reconstructions in Supplementary Fig. 6b). In addition, we found that imposing rotation invariance further deteriorates reconstruction of image models across all structures (Supplementary Fig. 4c). Once again, an alternative approach confirmed that poor reconstruction was not due to dataset issues (Supplementary Fig. 4d and ‘Masked autoencoders using vision transformers’ in Methods).

Both classical and rotation-invariant point cloud models produced more accurate and comparable reconstructions, but with spatial distribution artifacts for structures with fewer training samples, like endosomes and peroxisomes, like the classical image model (as shown by arrows in Supplementary Fig. 4f,g). In addition to providing improved reconstructions compared to image models, we found that the rotation-invariant point cloud representations performed well at both structure classification (~95% accuracy versus 90% accuracy for best image model) and cell-stage classification (~58% accuracy versus 57% accuracy for best image model), while being more compact and orientation independent (Fig. 4b).

**Extended Data Fig. 3:**
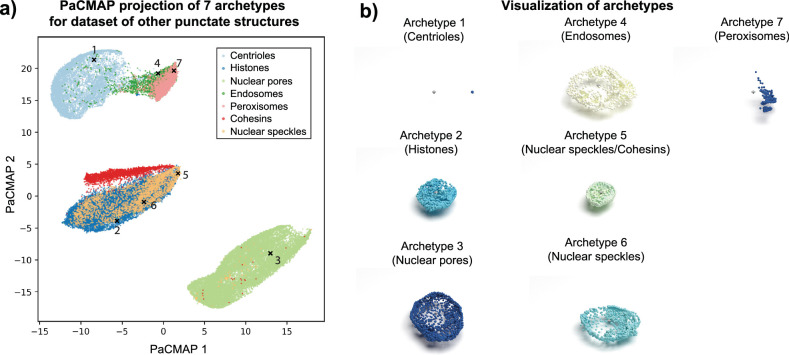
Archetype analysis for punctate structures from the WTC-11 hiPS Single-Cell Image Dataset v1. **a)** PaCMAP projection of the representations and archetypes colored by intracellular structure labels. **b)** 7 archetypes computed from the rotation invariant point cloud representations. Each archetype corresponds to one of the intracellular structures. Source data

We then fit PCA to the dataset of each structure independently to interpret the rotation-invariant point cloud representations. First, we sampled real single-cell images of each structure along PC1 as shown by the top and side views in Fig. 4c. From these images we were able to draw some observations. Figure 4c suggests the major source of variation across all seven intracellular structures in this dataset appears to be aspects of cell and nuclear shape like height and elongation. Next, we noticed that centrioles are localized near the nucleus at one extreme of PC1 and gradually migrate toward the cell membrane at the other extreme (see the column ‘Centrioles’ in Fig. 4c). Additionally, we observed nuclear speckles to be more uniformly distributed within the nucleus at one extreme of PC1 and more concentrated near the nuclear shell forming a ring-like pattern at the other extreme of PC1. Surprisingly, latent walks along PC1 revealed similar patterns of sources of variation for both centrioles and nuclear speckles compared to the original images (Fig. 4d). We found that centrioles polarize by moving away from the cell center (represented by a dark cross in Fig. 4d), and nuclear speckles concentrate in a ring-like pattern (last row of column ‘Nuclear speckles’ in Fig. 4d). To explore clustering in the representation space, we visualized all representations using a PaCMAP projection, coloring them by intracellular structure (Extended Data Fig. 3a). Nearly all structures formed distinct clusters, with some overlap observed between the clusters for nuclear speckles and histones. Next, we conducted an archetype analysis using seven archetypes and projected these into the PaCMAP space. Each archetype corresponded to one of the clusters. Using generative reconstructions, we visualized the archetypes and found that each captured the distinctive morphology of its associated structure (Extended Data Fig. 3b). Notably, this result was not guaranteed, as archetypes are mathematical points in the representation space and could theoretically capture any variable aspect of intracellular structure morphology. Overall, these results highlight the ability of our rotation-invariant point cloud representations to capture meaningful and biologically relevant variations in the spatial pattern of multiple intracellular structures.

### Generalizing the framework to polymorphic multi-piece structures

**Extended Data Fig. 4:**
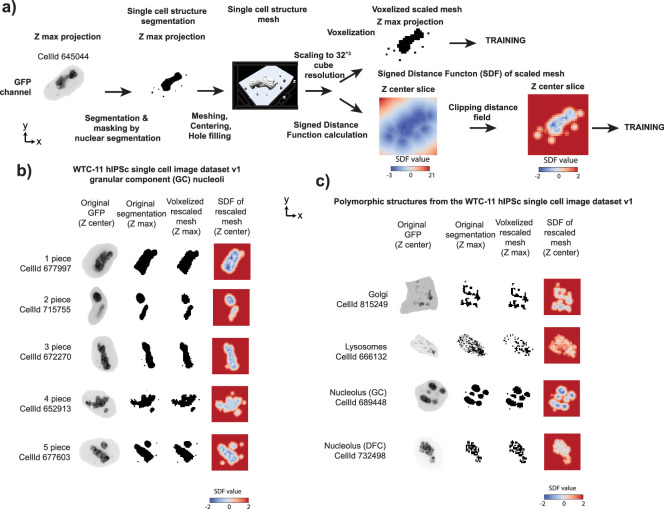
3D image preprocessing into application appropriate inputs for polymorphic structures. **a)** Workflow for computing signed distance function (SDF) images from segmentations. single-cell structure segmentations are masked by nuclear segmentation (for nuclear structures), followed by meshing, centering, and hole filling. The mesh is then rescaled to 32*^3^ cube resolution and then processed to get a signed distance function. Alternatively, the rescaled mesh is voxelized to get a segmentation. SDF is clipped to (-2, 2) range for training image models to focus models on the zero level set. Example shown is for nucleoli (GC). **b)**. Visualization of rescaled segmentation and SDF for examples with different numbers of pieces of granular component (GC) of nucleoli. Shown are center-slices of raw intensity images, max projection of the structure segmentation, max projection of the voxelized rescaled segmentation, and center slice of the rescaled mesh SDF. **c)** Visualization of rescaled segmentation and SDF for other polymorphic structures from the WTC-11 hiPSC Single-Cell Image Dataset v1 including lysosomes, Golgi, GC nucleoli, and dense fibrillar component (DFC) nucleoli.

We next asked if we could adapt our approach to learn 3D rotation-invariant representations for non-punctate intracellular structures, such as nucleoli and the Golgi apparatus. These organelles are polymorphic structures where the shape of individual pieces, in addition to the location of these pieces, may be important for the underlying biological process and, therefore, should be captured by the learned representations^21,22^. We combined the point cloud approach with an SDF^11,23,24^ computed from segmented images to incorporate the shape information of individual pieces into the representation learning framework (Fig. 1c, Extended Data Fig. 4, ‘Polymorphic structure datasets’ in Methods and Supplementary Note 6).

**Fig. 5:**
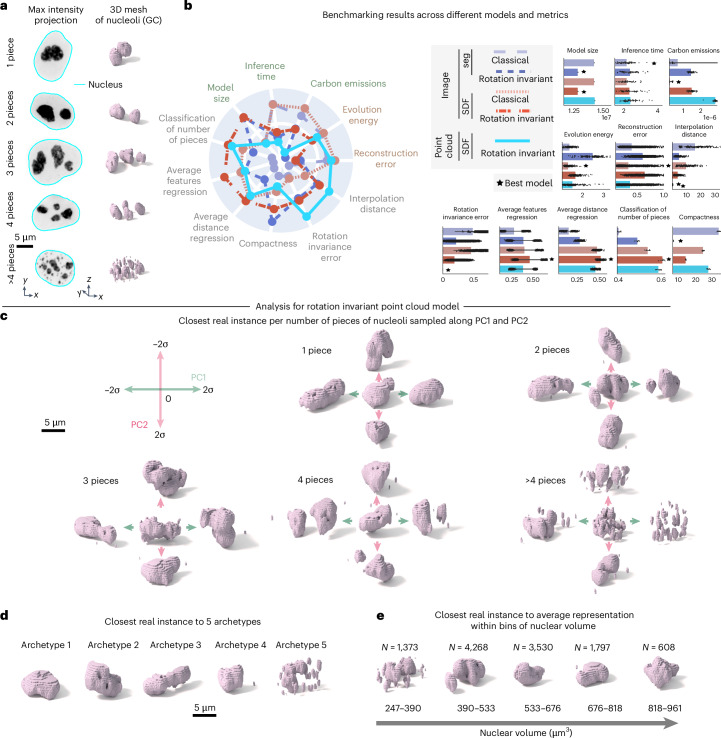
Rotation-invariant representation learning framework generalizes to polymorphic multi-piece structures. **a**, Dataset of nucleoli (GC) from the WTC-11 hiPS cell single-cell image dataset (v1)^1^, stratified by number of pieces. Shown are example maximum intensity projections and corresponding 3D meshes. **b**, Benchmarking unsupervised representations across different models and metrics. Left, Polar plot showing the performance of all models across efficiency metrics (model size (*n* = 1), inference time (*n* = 40) and emissions (*n* = 40)), generative metrics (reconstruction (*n* = 1,773) and evolution energy (*n* = 180)) and representation expressivity metrics (compactness (*n* = 5), classification of number of pieces (*n* = 5), shape features regression (*n* = 100), distance features regression (*n* = 100), rotation invariance error (*n* = 7,092) and average interpolation distance (*n* = 180)). Metrics are *z*-scored and scaled such that larger is better. Right, bar plots showing raw metric values across models for each metric. Error bars are the s.d. The best model for each metric is indicated. **c**, PC1 and PC2 for 1 piece, 2 pieces, 3 pieces, 4 pieces and >4 pieces as examples using the rotation-invariant point cloud model. PCA is fit to representations of different numbers of pieces separately. Shown are the closest real examples to normalized PCs (s.d./*σ*) sampled at three map points (−2σ to 2σ in steps of σ). **d**, Closest real example to five archetypes identified using the rotation-invariant point cloud representations. **e**, Closest real example to average representations of five equally sized bins of nuclear volume (Supplementary Note 5.4). Source data

We applied the adapted framework to images of the granular component (GC) of nucleoli via fluorescently tagged nucleophosmin (NPM1, *N* = 11,814; Fig. 5 and ‘WTC-11 hiPS cell single-cell image dataset v1’ in Methods), which are part of the WTC-11 hiPS cell single-cell images dataset (v1)^1^. Nucleoli are multi-compartment condensates that exhibit a broad distribution in both the number of pieces and size^25^ and can exhibit rapid rotation in 3D^26,27^. Given these properties, we expected that 3D rotation-invariant representations learned using an implicit definition of the nucleolar surface via an SDF would be more interpretable than representations learned by classical models directly from segmented images. To evaluate this, we trained two classical image models using segmentations and SDFs, two 3D rotation-invariant image models using segmentations and SDFs and one 3D rotation-invariant point cloud model using SDFs (see ‘Polymorphic structures’, ‘Polymorphic structure datasets’ and ‘Models’ in Methods). Examples of inputs and outputs of each of these models can be seen in Supplementary Fig. 5. All models were trained on downsampled images by scaling the meshes down to a resolution of 32 × 32 × 32 (Extended Data Fig. 4a). This rescaling process retained much of the relevant nucleolar information based on visualization (compare voxelized rescaled mesh and original segmentation in Extended Data Fig. 4b).

We found that the two classical image models based on segmentations and SDFs, and the 3D rotation-invariant point cloud model generate similar quality reconstructions (‘Reconstruction error’ in Fig. 5b). However, the point cloud model was less efficient in terms of emissions and inference time (Fig. 5b). We also found that 3D rotation-invariant image models produce lower quality reconstructions compared to classical image models, as we had observed for models trained on punctate structures (see Supplementary Note 1.1 for details on how reconstruction error was computed for each model). The results also indicate rotation-invariant representations from point clouds are more orientation independent compared to representations learned from both segmentations and SDFs (‘Rotation invariance error’ in Fig. 5b). Next, we asked which representations would capture more relevant morphological attributes of nucleoli. To answer this question, we used the learned representation to classify the number of nucleolar pieces in the segmented images and to predict the size, surface area and relative distance between pieces (Supplementary Note 1.2). We found that rotation-invariant image SDF and point cloud representations performed best on all these tasks (‘Classification of number of pieces’, ‘Average feature regression’ and ‘Average distance regression’ in Fig. 5b), suggesting that these representations contain relevant biological information. We also downscaled the meshes to a resolution of 64 × 64 × 64 to test the effect of downsampling the data to a resolution higher than the 32 × 32 × 32 resolution used before (Supplementary Fig. 6a). We found that the quality of the learned representations was similar, at the cost of higher emission scores (Supplementary Fig. 6b). Overall, we observed that no single model performs well across all metrics, thus requiring application-appropriate model selection. We observed similar results with a larger dataset of other polymorphic structures (Supplementary Note 7 and Extended Data Fig. 6). In both cases, we prioritized rotation invariance error as a representation expressivity metric, and reconstruction loss as a generative metric for downstream analysis (Supplementary Note 1).

Next, we used PCA on data grouped by number of nucleolar pieces per cell to interpret the rotation-invariant point cloud representations, with PCA fit separately for each group. Since we had to relax the generative capabilities of this model to achieve rotation invariance, we retrieved the closest real cells while performing a latent walk of PC1 and PC2 (Fig. 5c). We found elongation to be the major source of variation for single-piece nucleoli (~30% of the examples in the dataset; *N* = 3,499, explained variance of PC1 was 16% and PC2 was 7%). This was confirmed by computing the Pearson correlation with structure elongation (*r* = 0.56 for PC1). In the remaining 70% of the dataset (*N* = 8,315), where nucleoli consist of multiple pieces, the predominant source of variation appears to be the distance between pieces and the relative size of these pieces. For example, when considering nucleoli composed of two pieces, we observe PC1 (explained variance was 19%) to represent the height of the larger piece and the size of the small piece (Fig. 5c). In addition, we found PC1 to correlate with the average distance between pieces (*r* = 0.42 for PC1). By performing an archetype analysis with five archetypes on nucleoli representations from the entire dataset, we found that three archetypes represent nucleoli with a single piece but different elongations (archetypes 1–3 in Fig. 5d). Archetype four represents nucleoli with one large piece and one small piece, which is a common configuration in the dataset, and archetype five represents nucleoli fragmented in many small pieces.

**Extended Data Fig. 5:**
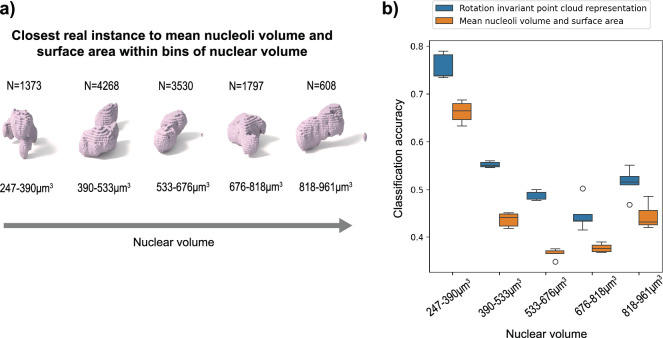
Comparison of rotation invariant point cloud representations of nucleoli (GC) to mean nucleolar volume and surface area. **a)** Closest real example to mean nucleolar volume and surface area within five equal sized bins of nuclear volume (*Section 7.3 of Methods*). **b)** Classification of number of nucleolar pieces across bins of nuclear volume using rotation invariant point cloud representations and mean nucleolar volume and surface area. Source data

Motivated by previous observations of cell-cycle-dependent nucleolar morphology^28^, we asked whether rotation-invariant representations could capture nucleolar changes as a function of the cell cycle. To do this, we again used nuclear volume bins to create a pseudo cell-cycle axis. Next, we computed an average nucleolar representation for increasing nuclear sizes and then visualized the closest real example to the average representation within each bin (Supplementary Note 5.3). Consistent with previous observations, we found that cells exiting division (small nuclear volume) have nucleoli that are fragmented into multiple pieces that coalesce into fewer larger pieces as the cells grow and progress toward mitosis (Fig. 5e). As a baseline for comparison, we also calculated the mean nucleolar volumes and surface areas for each nuclear volume bin and retrieved the closest real example for each group based on this feature set (Extended Data Fig. 5a). As a result of this analysis, we no longer observed a clear transition from fragmented nucleoli to single-piece nucleoli. We also quantified classification accuracy for the number of nucleolar pieces using mean nucleolar volumes and surface areas. We observed that this feature set performs worse than the learned representations (Extended Data Fig. 5b and Supplementary Note 5.4). Altogether, the results show that this representation learning framework can be successfully adapted using SDFs to polymorphic structures and that it provides representations that capture relevant aspects of the nucleolar biology.

### Evaluating drug effects on nucleolar morphology

We proceeded to test the applicability of the representation learning approach to a perturbation detection task using a drug screening dataset. We imaged WTC-11 hiPS cells expressing an endogenously, fluorescently tagged nucleophosmin, representing the GC of nucleoli. Cells were treated with 16 different drugs at relatively low concentrations to induce subtle phenotypic alterations (‘Drug dataset’ in Methods). Analysis was conducted on cells imaged 2 h after treatment.

We used the representation learning framework to extract unsupervised representations for cells in the dataset (*N* = 1,025). To do so, we fine-tuned the models trained on the dataset of nucleolar (GC) single-cell images described in the previous section. We followed the methods described by Chandrasekaran et al.^2^ to evaluate the performance of these fine-tuned models. This evaluation involved computing the mean average precision to measure how distinguishable different single cells of a drug-treated set are from untreated cells (dimethylsulfoxide (DMSO); *N* = 140), and a *q*-value statistic based on permutation testing. We included two baseline models to obtain a set of reference *q* values. To validate our morphological profiling approach, we first benchmarked performance using 3D CellProfiler features on nucleolar (GC) single-cell segmentations (Supplementary Note 8). Second, we assessed the generalizability of our SDF point cloud model by evaluating its performance on the nucleolar perturbation dataset without fine-tuning, treating it as an external validation set.

**Fig. 6:**
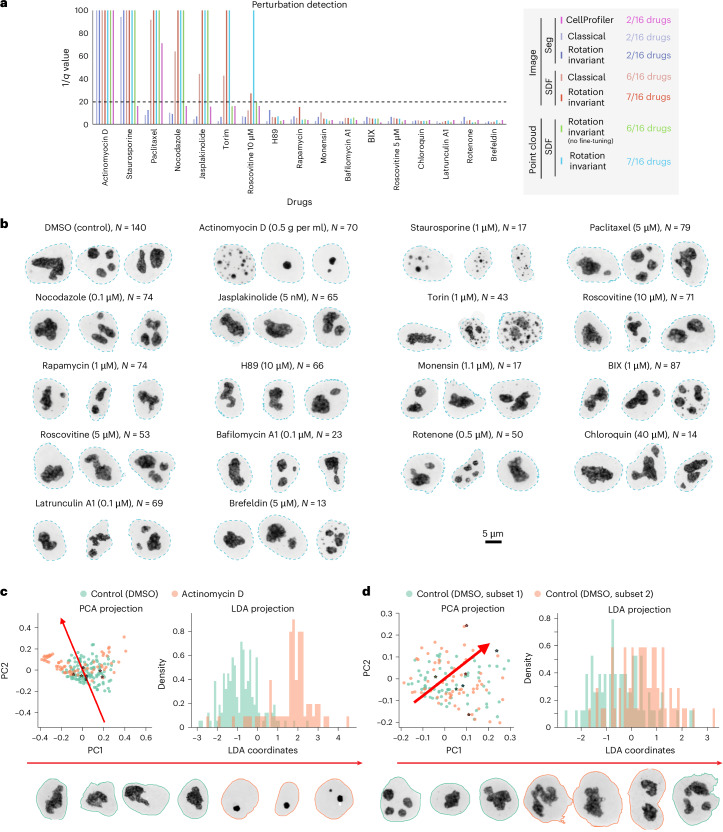
Learned representations allow for morphological profiling of nucleoli under different perturbations. **a**, *q*-value statistics per drug (‘Drug dataset’ of Methods) and per model indicating the confidence of each model distinguishing a given drug from control. Bar plots show 1/*q* for each model and drug. The *y*-limit is set to 100 to highlight the range of values around the 0.05 confidence threshold (dashed line at 1/*q* = 20). **b**, Three representative examples of nucleoli (GC) for the control (DMSO) and each of the 16 drugs used in this study. **c**,**d**, LDA analysis for the rotation-invariant point cloud representations of nucleoli for actinomycin D (left; *n* = 210) and a baseline using two random subsets of plates from the DMSO control (right; *n* = 140; Supplementary Note 5.5). Source data

The results are summarized in Fig. 6a, where we plot the *q* value per drug for each model. Drugs with *q* value under the significance threshold of 0.05 (or 1/*q* value > 20) are considered by that model as causing alterations in nucleolar morphology. Aside from the first two drugs, we found a difference in the behavior of CellProfiler, segmentation-based and SDF-based models. Therefore, we sorted the *x* axis from low to high *q*-values averaged over all SDF models. Consequently, drugs on the left side of the plot induce a stronger phenotypic change compared to drugs on the right side. Extended Data Table 1 describes details about each drug, such as name, concentration, molecular target or mechanism of action, effect based on literature review and effect observed on nucleoli based on visual inspection of this drug dataset. Representative examples of the range of phenotypes of each drug are shown in Fig. 6b.

The first drug to appear on the *x* axis of Fig. 6a is actinomycin D, indicating that this drug is the one with the strongest effect on nucleolar morphology. This drug works as a control in this analysis because it is the only well-characterized drug in this study that is known to target DNA and cause an alteration in nucleolar size (Extended Data Table 1 and Fig. 6b). Next, we found staurosporine to have the second strongest effect. A visual inspection of images of cells treated with this drug revealed the presence of many dead cells where nucleoli display very abnormal morphology (Fig. 6b). Both actinomycin D and staurosporine were identified as being distinguishable from the control (below *q* = 0.05 threshold) by all representation learning models. Interestingly, the CellProfiler features did not identify staurosporine, indicating that the representation learning models provide an improvement compared to this ‘classic’ baseline.

No other drug was identified by either the classical or rotation-invariant image-based segmentation models. On the other hand, the SDF-based models identified several other drugs that could be associated with off-target effects. Starting from the left side of the plot in Fig. 6a, these models next identified paclitaxel and nocodazole, which were associated with cell death 24 h after treatment, while also locking cells in mitosis (Extended Data Table 1 and Fig. 6b). SDF-based models also detected jasplakinolide, which is known to promote actin polymerization and is associated with higher nuclear volumes^29^. Visual inspection of the image data did not reveal any apparent nucleolar alteration (Fig. 6b) or change in cell health within 24 h after treatment, suggesting that the effect of this drug is subtle. Next, torin-2 was detected by all SDF models, which was observed by visual inspection to induce cell death, like staurosporine. Lastly, roscovitine at 10 µM was detected only by the SDF rotation-invariant models. While there was no visible alteration of nucleoli morphology in this dataset at this concentration, roscovitine at 10 µM is known to cause nucleolar segregation^30,31^ at higher concentrations. These results suggest that our representation learning framework captures subtle concentration-dependent phenotypes that are not visible by eye. Interestingly, the SDF rotation-invariant model trained on the nucleolar (GC) images from the WTC-11 hIPS cell single-cell image dataset v1 was also able to identify these drugs as being distinguishable from the control, despite never having seen any of the images in the perturbation set, suggesting that this model is able to generalize well to different conditions. Overall, we observed three different categories of drugs that were retrieved using different models: ‘sledgehammer’ phenotypes that were detected by all models, subtle off-target effects that were detected by SDF models, and subtle concentration-dependent phenotypes that were detected only by the rotation-invariant SDF models. The remaining nine drugs in the dataset did not induce visible alterations to nucleolar morphology, although four of them induced cell death at later time points, including H89, chloroquine, rotenone and brefeldin.

**Extended Data Fig. 6:**
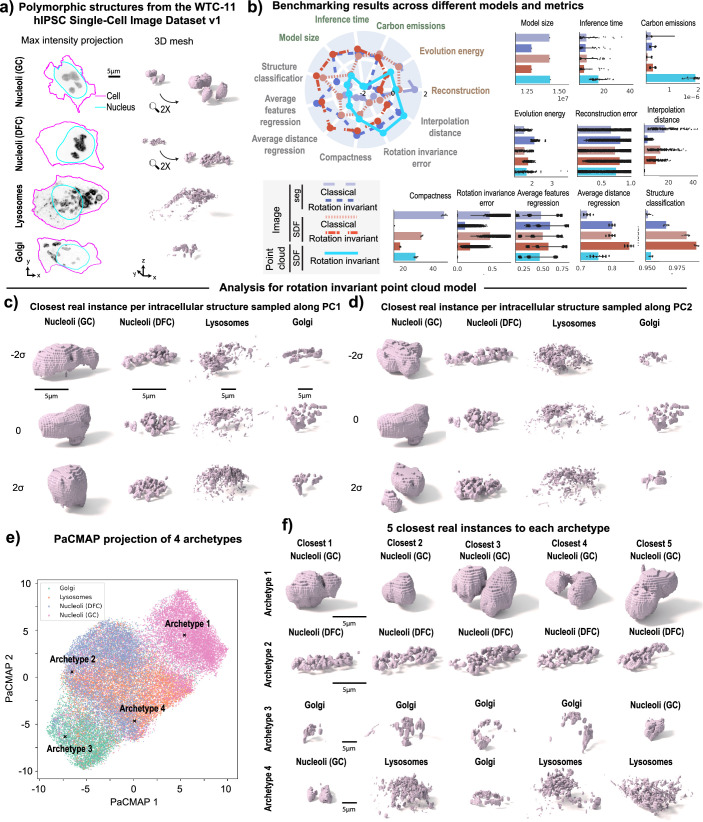
Learned representations enable shape variation profiling on multiple polymorphic structures. **a)** Dataset of nucleoli GC, nucleoli DFC, lysosomes, and Golgi from the WTC-11 hiPSC Single-Cell Image Dataset v1 (Viana 2023). Shown are example max intensity projections and corresponding 3D meshes. **b)** Benchmarking unsupervised representations across different models and metrics. (Left) Polar plot showing performance for all models across efficiency metrics (model size (n = 1), inference time (n = 40), emissions (n = 40), generative metrics (reconstruction (n = 5,706), evolution energy (n = 180)), representation expressivity metrics (compactness (n = 5), classification of number of pieces (n = 5), shape features regression (n = 100), distance features regression (n = 100), rotation invariance error (n = 16,005), average interpolation distance (n = 180)). Metrics are z-scored and scaled such that larger is better (Right) Bar plots showing raw metric values across models for each metric. Error bars are standard deviations. Best model for each metric is indicated. **c)** 1st principal component for each structure using rotation invariant point cloud model. PCA is fit to representations of each structure separately. Shown are closest real examples to normalized PCs (standard deviation (s.d.), σ, units) sampled at 3 map points (-2σ to 2σ in steps of σ). **d)** 2nd principal component for each structure using rotation invariant point cloud model. Shown are closest real examples to normalized PCs (standard deviation (s.d.), σ, units) sampled at 3 map points (-2σ to 2σ in steps of σ). **e)** PaCMAP projection of four archetypes and all representations for the rotation invariant point cloud model. **f)** 5 closest real instances to each archetype using the rotation invariant point cloud model. Source data

To further interpret the retrieved hits, we performed linear discriminant analysis (LDA)^1^ using the learned rotation-invariant point cloud representations (Fig. 6c, Extended Data Fig. 6 and Supplementary Note 5.5) to identify examples along this ‘axis of phenotypic difference’. Specifically, we sampled real cells along the principal LDA direction for each control–drug pair. We found the retrieved cells to be interpretable for most drug treatments. In the case of sledgehammer phenotypes like actinomycin D, staurosporine and torin, we observed a transition that recapitulated visual observations (Fig. 6c and Extended Data Fig. 6). For other drugs that were potentially associated with off-target effects, we observed changes in the number of pieces of nucleoli (paclitaxel and nocodazole), the size of nuclei and nucleoli (jasplakinolide) and both the size and number of pieces of nucleoli (roscovitine at 10 µM; Extended Data Fig. 6). As a baseline, we observed no phenotypic differences between random subsets of the control dataset using the same analysis (Fig. 6d and Supplementary Note 5.5). Overall, these results illustrate that our 3D representation learning approach can enable perturbation detection and phenotype profiling and indicate the need for follow-up experiments to confirm the impact of some of these drugs, like jasplakinolide, on nucleolar morphology.

## Discussion

In this paper, we developed a morphology-appropriate 3D rotation-invariant representation learning framework for multi-piece intracellular structures using point clouds. We benchmarked this framework against classical and rotation-invariant image-based models using a new multi-metric evaluation criterion that focuses not only on traditional reconstruction quality but also on measurements that can be important for downstream analysis and biological discovery. We found that application-appropriate model selection can be key to achieving optimal results, and that a morphology-appropriate approach can lead to more compact and expressive representations across a range of tasks when compared to classical image models. We applied this framework to synthetic and real single-cell image datasets for punctate structures, like DNA replication foci, and polymorphic structures, such as nucleoli. Our results reveal that geometry-aware choices of encodings and neural network architectures can enable unsupervised discovery and interpretation of variability in the morphology of several multi-piece intracellular structures. For example, the learned representations for the centriole capture its repositioning from the cell center toward the periphery, a behavior that is known to be mediated by the microtubule network^32,33^. The learned representations also recapitulate a known axis of morphological change of nuclear speckles, which goes from many, small, irregularly shaped speckles to larger, rounder shaped speckles. This is known to occur when transcription is inhibited in cells and is also the primary axis of variability between cell types^34^. We further evaluated the utility of our approach on phenotypic profiling of a nucleoli-perturbed image dataset and demonstrated the interpretability of the learned representations. In general, we noticed that segmentation-based models were not able to detect drugs like torin-2 that caused clear alterations on nucleolar morphology because of cell death. This result suggests that SDF encodes information relevant for perturbation detection. Additional discussion is available in Supplementary Note 9.

A key result from our analysis on polymorphic structures is that no single model performs well across all metrics. This is related to the sparsity–reconstruction trade-off where a model that perfectly reconstructs the input can learn a complex and entangled set of features, whereas a model that learns a sparse and disentangled set of features can reconstruct poorly^35^. In the cases where there is a trade-off, the choice of the best metrics to represent the best model can be application dependent. In this study, we prioritized rotation invariance error as a representation expressivity metric, and reconstruction loss as a generative metric. More generally, for tasks like production-scale drug screening, a scalable and efficient model that is predictive of different drug signatures may be more relevant. For tasks like biological discovery in small datasets, a model that learns compact and rotation-invariant features may be more interpretable. Finally, for tasks like generating virtual cell images of intracellular structures, a model that reconstructs the data well may be crucial.

Our framework can be further improved in multiple ways. For example, our results indicate cell and nuclear shape are major sources of variation because that information was not factored out of our learning framework and, therefore, become confounding variables. While this reflects a true coupling between cell and nuclear shape and structure localization, alternative approaches may offer a way to decouple these confounding variables from learned representations. For instance, one could incorporate reference information about other intracellular structures for answering questions about intracellular structure colocalization^35,36^. Another possibility for improving the proposed framework could be via incorporating multichannel information for multiple structures that have been simultaneously tagged^37,38^. By calculating the SDF based on the presence of multiple structures, one could compute a single composite field that contains information about all structures. This would implicitly encode mutual exclusivity rules, thus helping to further constrain the models and move toward a better understanding of compartmentalization^15^. We could also use multichannel information to extend the framework to predict spatial patterns of a set of structures given the representation from another set, thereby synthetically combining the reconstructions of different structures into one. For example, we can learn a shared representation across DNA replication foci and nucleoli images and leverage this shared representation to predict nucleolar morphology in a dataset of DNA replication foci. Such approaches could be used to build a holistic description of intracellular organization. Finally, to further improve interpretability and move toward mathematical descriptions of the learned representations, we can use symbolic regression methods like PySR^39^ to extract equations and summarize the quantitative model. It would also be particularly exciting to leverage time-series datasets to learn dynamical systems and extract biophysical measurements like rigidity. In all cases, incorporating additional information during the learning process can potentially make the models more robust and interpretable. In this work, we have focused on benchmarking unsupervised methods for datasets where a user has limited prior knowledge to establish a baseline. However, the morphology-appropriate representation learning using point clouds and SDFs that we have described here is flexible and can be modified to incorporate several such improvements.

In summary, we have begun to develop a computational analysis pipeline for interpretable representation learning of complex multi-piece intracellular structures. An important goal of this work is to make the data, models and analysis tools freely available to the community, so that it can serve as a benchmark for further development of methods for 3D analysis (‘Code availability’). We hope that this work can spur the interest of the cell biology community into new ways of analyzing and interpreting complex intracellular organization.

## Methods

### Single-cell image datasets

#### DNA replication foci dataset

Spinning-disk confocal 3D images taken of a fluorescently tagged cell line that targets PCNA labeling DNA replication foci with mEGFP were processed to create the DNA replication foci dataset^40^. Fluorescent cell membrane and DNA dyes tagged the cell boundary and nucleus, respectively. Nuclear segmentations were obtained using the protocol described by Viana et al.^1^, with the only difference being that nucBlue dye was replaced with nucViolet dye. Segmentations of DNA replication foci were generated for each field of view (FOV), using three different segmentation workflows created using the ‘Allen Cell & Structure Segmenter’^41^ to segment specific DNA replication foci morphologies. Next, we visually identified which segmentation workflow was best for each cell and saved the result in an empty FOV at that cell’s correct location. More details about the dataset and images are available here https://open.quiltdata.com/b/allencell/packages/aics/nuclear_project_dataset_4/.

Cells in interphase were labeled by an expert as belonging to one of nine classes—G1, early S, early–mid S, mid S, mid S–late S, late S, late S–G2, G2 and unclear. Unclear labels were dropped during analysis. About 3% of cells were labeled as outliers based on bad segmentations of DNA replication foci, cells appearing dead or dying, no EGFP fluorescence and bad segmentations of cells and nuclei. Dead cells and no fluorescence were used for the outlier detection task, accounting for 16 cells of a total of 2,420 cells.

#### WTC-11 hiPS cell single-cell image dataset v1

Spinning-disk confocal 3D images taken from 25 endogenously tagged hIPS cell lines were processed to create the WTC-11 hiPS cell single-cell image dataset (v1)^1^. Fluorescent cell membrane and DNA dyes tagged the cell boundary and nucleus, respectively. Cell, nuclear and structure segmentations were used as provided in the dataset release available at https://open.quiltdata.com/b/allencell/packages/aics/hipsc_single_cell_image_dataset/.

We performed analysis on histones via H2B (*N* = 15,875), nuclear pores via Nup153 (*N* = 17,703), peroxisomes via PMP34 (*N* = 1,997), endosomes via Rab-5A (*N* = 2,601), centrioles via centrin-2 (*N* = 7,575), cohesins via SMC1A (*N* = 2,380) and nuclear speckles via SON (*N* = 2,980) as selected punctate structures from this dataset. We selected nucleoli (DFC) via fibrillarin (*N* = 9,923), nucleoli (GC) via nucleophosmin (*N* = 11,814), lysosomes via LAMP-1 (*N* = 10,114) and Golgi via sialyltransferase (*N* = 6,175) as polymorphic structures. While we used all single-cell images for training our models, we limited our analysis to interphase cells.

#### cellPACK synthetic single-cell dataset

We used cellPACK to create synthetic point clouds within real nuclear shapes^17^. cellPACK provides an algorithm to create high-resolution 3D representations of the biological mesoscale based on specified rules. Segmentation of 254 randomly chosen nuclei from the DNA replication foci dataset were converted into a triangulated mesh and used as input to cellPACK. Here, the nuclei were pre-aligned to their longest axis. cellPACK then packed 256 spheres with a radius of 1 voxel within these meshes based on four distinct rules: (1) Random: points were generated uniformly at random inside the nucleus; (2) Planar gradient rule: points were generated inside the nucleus with a bias away from a plane. The plane contains the centroid of the nucleus, and its orientation is specified by a normal vector. We used normal vectors with three different orientations: (i) *θ* = 0, the normal vector points along the *z* axis (0*x* + 0 *y* + 1*z*) where the longest axis of the nucleus is the *y* axis. (ii) *θ* = 45°, the normal vector is (0*x* + 1/√2 *y* + 1/√2*z*). (iii) *θ* = 90°, the normal vector points along the *y* axis; (3) Surface gradient rule: points were generated with a strong bias toward the nuclear surface; (4) Radial gradient: points were generated with a bias toward the centroid of the nucleus. For each rule, cellPACK generated a point cloud with 256 points for each nucleus shape. This dataset is available for download at https://open.quiltdata.com/b/allencell/tree/aics/morphology_appropriate_representation_learning/cellPACK_single_cell_punctate_structure/.

#### Drug dataset

A collection of well-characterized drugs was used to perturb the Allen Institute for Cell Science cell line AICS-50 (WTC-11 hiPS cell endogenously tagged for mEGFP-NPM1, tagging nucleoli (GC)). Drugs and concentrations were selected because cell treatment with each of them induced a well-characterized effect on one major cellular structure morphology that could be visually observed within 24 h of treatment (Supplementary Table 1) and was not associated with massive cell death within the first 2 h of treatment, except for jasplakinolide. Cells were seeded on a 96-well glass-bottom plate using the protocol described by Gregor et al.^42^. Four days after seeding two-dimensional (2D) bright-field low-magnification well overviews were acquired and used for position selection following the same criteria as described by Viana et al.^1^. Following position selection, cells were washed once with pre-warmed phenol red-free mTeSR, and then medium was replaced with drug-containing phenol red-free mTeSR medium at the indicated concentration (Supplementary Table 2). The cells were then placed back on the spinning-disk confocal microscope stage where they were maintained at 37 °C with 5% CO_2_ for 2 h before the start of imaging at high magnification (×120). Images were acquired with three identical Zeiss spinning-disk confocal microscopes with a ×10/0.45 NA Plan-Apochromat (for well overview and position selection) and a ×100/0.8 NA Plan-Apochromat (Zeiss; for high-resolution imaging) and ZEN 2.3 software (blue edition; Zeiss). The spinning-disk confocal microscopes were equipped with a ×1.2 tube lens adapter for a final magnification of ×12 or ×120, respectively, a CSU-X1 spinning-disk scan head (Yokogawa) and two Orca Flash 4.0 cameras (Hamamatsu). 3D FOV image stack acquisition was performed with two cameras allowing for simultaneous acquisitions of a bright-field and an mEGFP (excited with 4.6 mW of a 488-nm laser) channel. The exposure time was 100 ms. The resulting images were of 16 bits and 924 × 624 pixels in the *xy* dimension after 2 × 2 binning. FOVs had a final *xy* pixel size of 0.108 μm and *z*-stacks composed of 100 *z*-slices (to encompass the full height of the cells within an FOV) acquired at a *z*-interval of 0.29 μm. Transmitted light (bright-field) images were acquired using a red LED light source with a narrow range peak emission of 740 nm and a BP filter of 706/95 nm for bright-field light collection. A Prior NanoScan Z 100-mm piezo *z*-stage (Zeiss) was used for fast acquisition. Optical control images of the field of ring (Argolight) and dark current were acquired daily at the start of each data acquisition to monitor microscope performance. Laser power was measured monthly, and the corresponding percentage was adjusted to consistently expose the sample to the same laser power. This dataset is available for download at https://open.quiltdata.com/b/allencell/tree/aics/NPM1_single_cell_drug_perturbations/.

##### Cell health assessment

We assessed cell health at 4 h and 24 h after drug treatment using for each drug both the AICS-57 (WTC-11 hiPS cell endogenously tagged for mEGFP-NMP1) and AICS-61 (WTC-11 hiPS cell endogenously tagged for mEGFP-HIST1H2BJ) cell lines. FOVs of this cell line were visually inspected to determine the extent of cell death induced by each drug. If cell death at either 4 h or 24 h was approximately 50% more prevalent than compared to the control, then cells were classified as unhealthy after 2 h. Otherwise, cells were classified as healthy. Results from this assessment are summarized in the last column of the table shown in Fig. 6b.

### Input data preprocessing for image models

#### Punctate structures

##### cellPACK synthetic dataset

Packing results were voxelized into images of 238 × 472 × 472 voxels in size. The *z*-coordinate of these images was padded with zeros to be the same size as *X* and *Y*, and the resulting images were downsampled to 118 × 118 × 118 voxels via block reduce operation with a block size of 4 × 4 × 4 voxels and then used as input for image-based models.

##### DNA replication foci dataset

3D raw fluorescence intensity single-cell images of DNA replication foci were masked, centered and aligned by the corresponding nuclear segmentation dilated by 8 × 8 × 8 voxels. Images were cropped and then padded to the largest nuclear bounding box in the dataset. Images were then padded and resized to 118 × 118 × 118 voxels. Images were globally contrast adjusted to be within the intensity range of 0 to 6,000, which was empirically determined to remove dead pixels present in a few images and scaled per image using min–max normalization via ‘monai.transforms.ScaleIntensity’^43^ to be in the range of (0, 1).

##### Expanded dataset of punctate structures

Similar preprocessing was applied to a subset of punctate structures from the WTC-11 hiPS cell single-cell image dataset (v1)^1^, including DNA replication foci, histones, nuclear pores, nuclear speckles, cohesins, peroxisomes, endosomes and centrioles. However, the images of cytoplasmic structures (peroxisomes, endosomes and centrioles) were masked by the cell membrane segmentation, instead of nuclear segmentation. Images were contrast adjusted using structure-specific intensity ranges reported in ref. ^1^. Images were finally scaled per image using min–max normalization via ‘monai.transforms.ScaleIntensity’^43^ to be in the range of (0, 1). The preprocessing code used to generate this dataset is available at https://github.com/AllenCell/benchmarking_representations/tree/main/br/data/preprocessing/image_preprocessing/.

#### Polymorphic structures

##### Nucleoli (GC) dataset

Segmentations of nucleoli (GC) available in the WTC-11 hiPS cell single-cell image dataset v1 were masked by corresponding nuclear segmentations. We used a hole-filling algorithm to fill in holes in the segmented images that were then converted into 3D meshes for subsequent preprocessing. Meshes were downscaled to fit within a cube of size 32 × 32 × 32 voxels using a global scaling factor to preserve the relative scale of nucleoli in learned representations. For segmentation models, the downscaled meshes were voxelized to create binary images. For SDF models, the downscaled meshes were used to compute SDF images that were clipped to be in the range of (−2, 2).

##### Expanded dataset of polymorphic structures

Segmentation of the nucleolar GC, nucleolar DFC, Golgi and lysosomes (available in the WTC-11 hiPS cell single-cell image dataset v1) were masked by either nucleus or cell mask if the structure localizes to nucleus (nucleoli) or cytoplasm (Golgi and lysosomes). Subsequent preprocessing followed the expanded dataset of polymorphic structures, except that 3D meshes were downscaled on a per-cell basis based on the cell’s intracellular structure bounding box. This downscaling avoids losing small nuclear structures given the large bounding box of cytoplasmic structures. While this scaling strategy prevents us from comparing sizes across different intracellular structures, it helps preserve the resolution of structures occupying only a few voxels.

##### Perturbed nucleoli (GC) dataset

We used the Allen Cell & Structure Segmenter to segment raw fluorescence intensity FOVs of perturbed nucleoli (GC; ‘Drug dataset’). Nuclear segmentations for each FOV were produced by applying a UNet model trained on the WTC-11 hiPS cell single-cell image dataset v1 to predict 3D nuclear segmentations from bright-field images. We manually selected nuclear segmentations in each FOV that covered the corresponding entirety of the nucleoli signal. The selected masks were used to generate single-cell images, and they were processed as described in ‘Nucleoli (GC) dataset’.

### Input data preprocessing for point cloud models

#### Punctate structures

##### cellPACK synthetic dataset

The list of *N* = 256 centroids of spheres packed by cellPACK was extended to 2,048 points by adding a small jitter to each input point cloud eight times. This jitter was clipped at a value of 0.2, and the typical range of *XYZ* coordinates was −10 to 10. This was then used as the 3D point cloud input. To improve reconstruction quality, this augmentation process was repeated ten times for each input. Details regarding the jitter augmentation are described in Supplementary Note 3.

##### DNA replication foci dataset

We started by applying the same preprocessing used in the DNA replication foci dataset described above for image-based models, except for the last linear scaling step. We then sampled 4D point clouds from the raw intensity images in two stages. In the first stage, we converted the raw intensity values into probabilities. We did this by using an exponential function ${e}^{\lambda ({skewness}* {intensity})}$ to scale the intensities. Here, the skewness is a statistic that indicates deviation of a distribution from a normal distribution, and the coefficient $\lambda$ is an intracellular-specific scale factor that was empirically determined based on the visualization of sampled points from random images for each intracellular structure. For nuclear structures, we used $\lambda$ = 100, and for cytoplasmic structures we used $\lambda$ = 500. The goal of this function was to exponentially increase the probability of sampling points from higher intensity values, to prevent sampling from the background. The scaled intensities were then converted into probabilities using normalization via dividing by the sum. The full probability of sampling a point cloud from the intensity image was defined as

$$P=\frac{{e}^{\,\lambda ({skewness}* {intensity})}}{{\sum e}^{\lambda ({skewness}* {intensity})}}.$$

We used these probabilities to sample a dense point cloud with 20,480 points (with intensity as a fourth coordinate) as shown in Supplementary Fig. 4. These points were centered using the nuclear centroid for nuclear structures, and the membrane centroid for cytoplasmic structures. In the second stage, we sampled a sparse point cloud with 2,048 points from the dense point cloud randomly during training while keeping the intensity as a fourth coordinate. The intensity coordinate was scaled using a factor of 0.1 to match the magnitude of the spatial coordinates. This sparse point cloud was then scaled using a global scale factor of 0.1 to reduce the range of the loss values and was passed as an input to the encoder (Fig. 1b).

##### Expanded dataset of punctate structures

We started by applying the same preprocessing used in the expanded dataset of punctate structures described above for image-based models, except for the last linear scaling step. We again used an exponential function ${e}^{\lambda ({skewness}* {intensity})}$, with $\lambda$ = 100 for nuclear structures, and $\lambda$ = 500 for cytoplasmic punctate structures. The scaled images were then normalized to obtain a probability density. We followed the same procedure described above for DNA replication foci to sample point clouds for each of these punctate structures. The intensity coordinate was then normalized using structure-specific contrast ranges.

#### Polymorphic structure datasets

##### Nucleoli (GC) dataset

Two sets of point clouds were sampled from segmented images of polymorphic structures. To do this, the segmented images were first converted into meshes and rescaled to a resolution of 32 × 32 × 32 (Extended Data Fig. 4). The first point cloud was generated by sampling 32 × 32 × 32 points randomly from the surface of the mesh. Then, the second point cloud was generated by calculating SDF values for a random list of 32 × 32 × 32 query points sampled within a 32 × 32 × 32-unit grid. Here, the SDF is a function that represents the signed distance of a position to the nearest part of a shape. During training, the first point cloud was subsampled randomly to 8,192 points and passed as an input to the encoder. The second point with the SDF values was subsampled randomly to 20,000 points and was then used as input query points to the decoder to generate SDF predictions (Fig. 1c).

##### Expanded dataset of polymorphic structures

Each single polymorphic structure image underwent a similar process. First, a point cloud of 8,192 points was sampled from the corresponding 3D mesh. During training, an additional 20,000 points were sampled from the SDF volume, producing a 4D point cloud (SDF value + *XYZ* coordinates).

##### Perturbed nucleoli (GC) dataset

The perturbed nucleoli (GC) dataset followed the same sampling strategy. For each single-cell nucleoli (GC) image, an initial 8,192 point cloud was sampled from the 3D mesh generated as described in ‘Perturbed nucleoli (GC) dataset’. During training, another point cloud containing 20,000 points was drawn from the SDF volume, yielding a 4D point cloud (SDF value + *XYZ* coordinates).

### Models

#### Image models

To implement 3D rotation-invariant image autoencoders, we used image encoders equivariant to the group of 3D rotations (SO3 group) using R^3^ steerable kernels as described in Weiler et al.^44^ and implemented in the ‘escnn’ library^45^. Compared to conventional convolutions, R^3^ steerable kernels are equivariant under rotations in R^3^. We used scalar fields to learn invariant scalar features in R^3^, and vector fields to learn equivariant vector features in R^3^. We used vector features to reconstruct the 3D rotation matrix as described by Deng et al.^11^ and Winter et al.^46^.

We used seven layers of steerable kernels with an equal number of hidden scalar fields using trivial representations and vector fields using irreducible representations. Using a (filter, stride, kernel size) convention, the convolutions were (8, 1, 3), (16, 1, 3), (32, 2, 3), (64, 2, 3), (128, 2, 3), (512, 2, 3) and (*N*, 1, 1), where *N* was the size of the latent dimension. In the final layer we used *N* scalar fields and two vector fields. Each convolutional block also included a batchnorm and ReLU activation. We used average pooling in the last five layers and checked that this did not break equivariance (Supplementary Fig. 1). We spatially pooled the scalar embedding in the final layer to get the final *N* dimensional rotation-invariant latent embedding. We used a bottleneck size of 512 for polymorphic structures and 256 for punctate structures.

The decoding function was a conventional neural network (CNN) decoder with six layers of convolutions. We used upsampling blocks with a scale factor of 2 in between convolutions. Using a (filter, stride, kernel size) convention, the convolutions were (512, 1, 3), (256, 1, 3), (128, 1, 3), (64, 1, 3), (32, 1, 3) and (16, 1, 3). We rotated the canonical reconstruction with the rotation matrix computed from the vector representation. We used a cylinder mask using ‘escnn.nn.modules.masking_module.build_mask’ to mask reconstructions and reduce interpolation artifacts. We set the background value to 0 for segmentations, and 2 for clipped SDF images where the maximum value was 2 and positive values were located outside the object. We used the same settings with classical autoencoders by swapping out equivariant convolutions with regular convolutions and keeping other details the same.

##### Masked autoencoders using vision transformers

We also trained masked autoencoders using vision transformers^47^ in two stages as an alternative to the ‘vanilla’ autoencoders described above. We performed this training in two stages. First, we pretrained a masked autoencoder^48^ using a *ZYX* patch size of (2, 2, 2), a mask ratio of 0.75 and learnable positional embeddings. The encoder was made up of eight identical transformer blocks, each with four heads and an embedding dimension of 256. The decoder had two layers with eight heads and an embedding dimension of 192. We then used a second phase of training with a mask ratio of 0 (that is, all image patches are visible to the encoder) where we froze the masked autoencoder-trained encoder and trained a freshly initialized decoder to reconstruct the input image. We trained all models with a mean squared error loss.

#### Point cloud models

To implement 3D rotation-invariant point cloud autoencoders, we used a 3D rotation-equivariant point cloud encoder using vector neurons (VNs^11^), which lifts classical neurons to 3D vectors resulting in 3D vector representations. VN layers are equivariant to rotations by construction and have been shown to outperform other equivariant architectures for tasks like classification, segmentation and reconstruction. We incorporate VN layers into a dynamic graph conventional neural network (DGCNN)^49^ backbone for point cloud encoding. DGCNN uses network modules called EdgeConvs to perform CNN-like local neighborhood feature extraction. These EdgeConvs can be stacked to extract global features^49^. Dynamic graphs are computed by constructing *k*-nearest neighbor graphs on points. We used *k* = 20 based on previous works as a balance between computational complexity and local structure information^50^. We concatenated the cross-product of the neighbor features and input points as well as the input points themselves to the hidden representation. As described in ‘Input data preprocessing for point cloud models’, we included raw image intensity in addition to *XYZ* coordinates in some cases to generate 4D point clouds. This coordinate was included with the same vector orientation as the *XYZ* coordinates and thus remains equivariant under rotations in R^3^. For the cellPACK dataset, we used a 3D point cloud as input. We used six convolutional blocks where each block comprises a VN linear layer and a VN leaky ReLU layer. We collated intermediate outputs before a final one-dimensional convolution. We took the norm of the final vector embedding to get a rotation-invariant representation. We also trained classical point cloud autoencoders with DGCNN encoders as described by Vries et al.^50^, where VN linear and VN leaky ReLU layers are replaced with edge convolutions and ReLU layers.

##### Decoder for punctate structures

We reconstructed the rotation-invariant representation for punctate structures using a folding net decoder^51^. This decoder concatenates the latent embedding with source points sampled from a template shape and then applies two folding operations with ReLU activations interleaved in between to reconstruct a point cloud. We used a 2D plane as a template in all cases except for the cellPACK synthetic dataset, where a sphere was used as a template. Next, we used the learned rotation matrix from the vector embedding to reorient the canonical reconstruction. We optimized the model using an earth mover’s distance^52^.

### Reporting summary

Further information on research design is available in the Nature Portfolio Reporting Summary linked to this article.

## Online content

Any methods, additional references, Nature Portfolio reporting summaries, source data, extended data, supplementary information, acknowledgements, peer review information; details of author contributions and competing interests; and statements of data and code availability are available at 10.1038/s41592-025-02729-9.

## Supplementary information

Supplementary Information Supplementary Figs. 1–7, Supplementary Tables 1 and 2 and Supplementary Notes 1–9

Reporting Summary

## Source data

Source Data Fig. 2 Statistical source data.

Source Data Fig. 3 Statistical source data.

Source Data Fig. 4 Statistical source data.

Source Data Fig. 5 Statistical source data.

Source Data Fig. 6 Statistical source data.

Source Data Extended Data Fig./Table 3 Statistical source data.

Source Data Extended Data Fig./Table 5 Statistical source data.

Source Data Extended Data Fig./Table 6 Statistical source data.

Source Data Extended Data Fig./Table 7 Statistical source data.

Source Data Extended Data Fig./Table 8 Statistical source data.

## Figures and Tables

**Extended Data Fig. 7:**
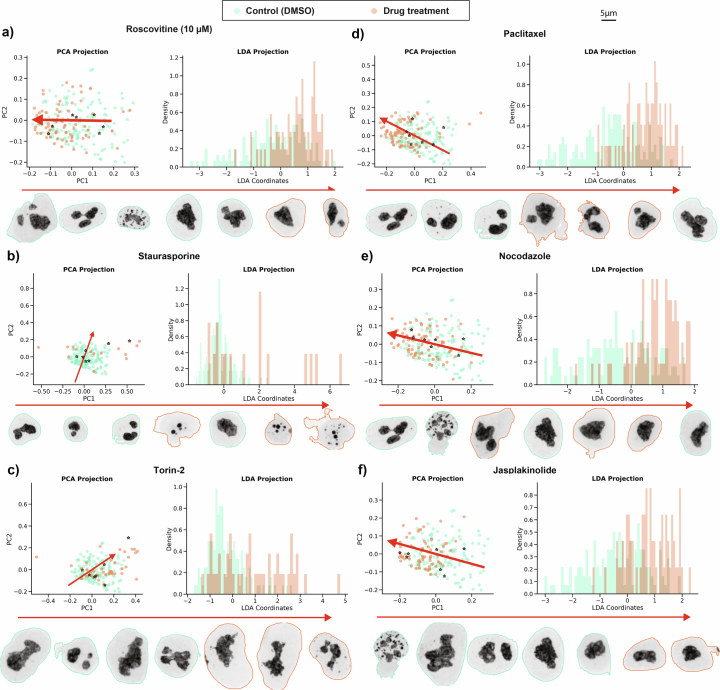
LDA analysis for rotation invariant point cloud representations of nucleolar drug perturbation dataset. Learned representations are reduced to 20 principal components (PCs) using PCA. Each drug is analyzed separately (**a** - **f**), together with the control (DMSO). For each drug, the left plot shows first two PCs with an arrow indicating the main discriminant axis that separates the control and the drug (left). Markers indicate the closest real cell along the LDA line computed in 20 dimensions. The right plot shows the density of the control and the drug along LDA coordinates. The bottom plot shows a max projection of the closest real cell sampled along the LDA line (*Section 7.5 of Methods*). Source data

**Extended Data Fig. 8:**
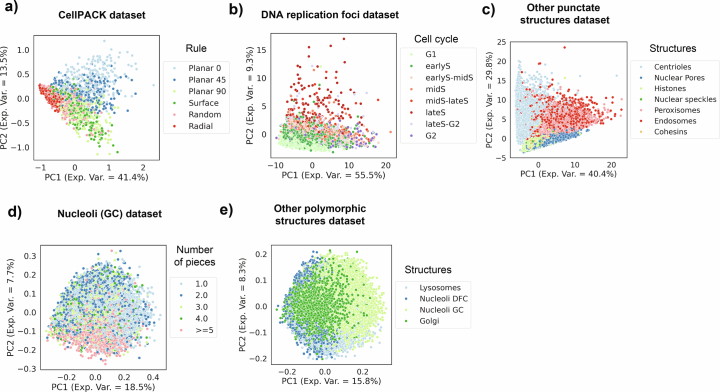
PCA score plots for all datasets. PCA fits using the representations of the rotation invariant point cloud models for the **a**) cellPACK dataset, **b**) DNA replication foci dataset, **c**) Other punctate structures from the WTC-11 hiPSC Single-Cell Image Dataset v1, **d**) Nucleoli (GC) from the WTC-11 hiPSC Single-Cell Image Dataset v1, and **e**) Other polymorphic structures from the WTC-11 hiPSC Single-Cell Image Dataset v1. Shown are PC1 and PC2 for all classes combined, and the points are color-coded according to the class they belong to. Source data

**Extended Data Table 1:**
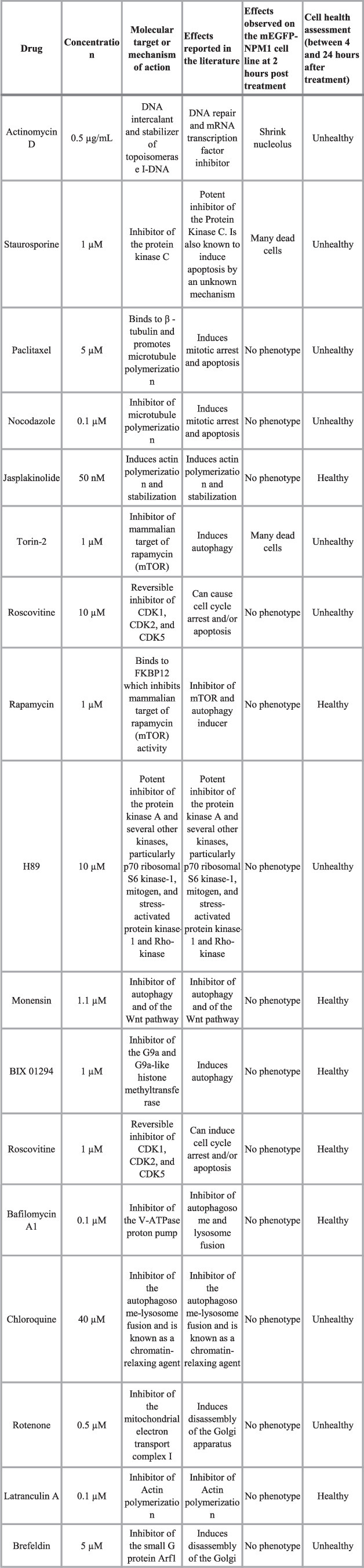
Drug descriptions

Name, concentration, molecular target or mechanism of action, effect based on literature review and effect observed on nucleoli of each drug based on visual inspection.

## Data Availability

The WTC-11 hiPS cell single-cell image dataset v1 analyzed in this study is available at https://open.quiltdata.com/b/allencell/packages/aics/hipsc_single_cell_image_dataset/. The DNA replication foci dataset analyzed in this study is available at https://open.quiltdata.com/b/allencell/packages/aics/nuclear_project_dataset_4/. The WTC-11 hiPS cell nucleoli (NPM1) perturbation single-cell image dataset analyzed in this study is available at https://open.quiltdata.com/b/allencell/tree/aics/NPM1_single_cell_drug_perturbations/. The synthetic dataset of punctate structures generated using cellPACK and analyzed in this study is available at https://open.quiltdata.com/b/allencell/tree/aics/morphology_appropriate_representation_learning/cellPACK_single_cell_punctate_structure/. The landing page of the GitHub repository associated with this paper (https://github.com/AllenCell/benchmarking_representations/) has additional information for accessing and processing these datasets. Source data are provided with this paper.

## Code availability

To aid reproducibility and empower researchers with the ability to independently test and apply these models to their own data, we provide all the representation learning models used in this study via CytoDL, a Python package for configurable 2D and 3D image-to-image deep learning transformations and representation learning, available at https://github.com/AllenCellModeling/cyto-dl/. CytoDL is designed consistent with FAIR^53^ practices, and is built to work for diverse use cases, thus making it robust, modular and flexible to the evolving nature of research.

Code to reproduce the representation learning models in this paper is available at https://github.com/AllenCellModeling/cyto-dl/blob/br_release/.

Config files associated with our models, training scripts, and code for multi-metric benchmarking are available at https://github.com/AllenCell/benchmarking_representations/. Our code was fully developed in Python. A list of dependencies is available at https://github.com/AllenCellModeling/cyto-dl/tree/br_release/requirements/ and https://github.com/AllenCell/benchmarking_representations/blob/main/pyproject.toml/. We have released the code with the Allen Institute Software License.
