# Supplementary material for: Interpretable representation learning for 3D multi-piece intracellular structures using point clouds
Source: Nat Methods. 2025 Jul 3;22(7):1531–44. doi: 10.1038/s41592-025-02729-9 (PMC12240800; doi:10.1038/s41592-025-02729-9)
Supplement: Supplementary file 1 — Supplementary Figs. 1–7, Supplementary Tables 1 and 2 and Supplementary Notes 1–9 [file 41592_2025_2729_MOESM1_ESM.pdf]

nature methods

Supplementary information https://doi.org/10.1038/s41592-025-02729-9

Interpretable representation learning for 3D multi-piece intracellular structures using point clouds

In the format provided by the authors and unedited

1. Multi-metric model evaluation

The multi-metric evaluation approach tested different models beyond their ability to reconstruct the input, which is the primary task of autoencoders. For example, this included efficiency metrics that gauged the compute resources and time required to evaluate each model, including the model size, inference time and carbon emissions. We also included generative metrics like the evolution energy between two sample shapes (Extended Data Fig. 1b). In addition, we also evaluated the models' expressivity by quantifying the biological information content in the learned representations. Specifically, we used representations for classifying or predicting (via regression) biologically relevant measurements in an application-appropriate manner. For instance, we used the representations to predict the volume or number of pieces of a given intracellular structure when these properties are relevant for the downstream application. Considering all these metrics together, we quantified the holistic utility of each model and the pros and cons of using each approach.

We compared the different metrics via z-scoring and visualization using a polar plot (Extended Data Fig. 1c). In all cases, we qualitatively determined the best model by picking the model that was consistently performing well across all metrics (Fig. 2, 3, 4). In the cases where we observed a trade-off with no single model performing well across all metrics, we picked the model that was performing well across the representation expressivity and generative metrics (Fig. 5, 6). Specifically, low reconstruction losses indicate the completeness of the representations, whereas low rotation invariance errors indicate the removal of information related to rotations. In the case of the nucleoli dataset and the rotation invariant SDF point cloud model, a combination of these two metrics highlights that the model can factor out information related to rapid rotations of nucleoli, while focusing on the rapid shape changes. Importantly, this model also captures relevant aspects of nucleolar morphology as shown via comparable regression and classification scores to the rotation invariant image SDF model (Fig. 5).

Efficiency: *Model size* is the number of parameters in the model, *inference time* is the time to run GPU inference on a single input, and *carbon emissions* is an estimate of hardware electricity power consumption in kWh. We evaluated inference time and carbon emissions on 40 test set examples given each modality and dataset to obtain error bars. While inference time and carbon emissions are related, inference time evaluates the time complexity of the algorithm, whereas carbon emissions evaluate both the time and spatial complexity of the algorithm. Higher memory consumption in the same amount of time will lead to higher carbon emissions. All measurements use a single A100 GPU.

Generative capacity: *Reconstruction error* is the average test set reconstruction error using the Jaccard similarity score for image segmentations and Chamfer distance for point clouds, respectively. Here, the

Jaccard similarity score is a metric used to evaluate the similarity of two binary sets/images. It is defined as the size of the intersection divided by the size of the union, as follows $J(A,B) = |A \cap B|/|A \cup B|$, where A and B are binary images. The chamfer distance is a metric used to evaluate the similarity of two point clouds. Given two point clouds A and B, it is defined as the average of the nearest distances from points in A to B and vice-versa. More details on classification and regression calculations can be found in section 1.1. *Evolution energy* is the normalized energy of deformation from one shape to another averaged across many random test set pairs in a dataset (Extended Data Fig. 1b). Here, the normalized energy of deformation is computed as the sum of the reconstruction error between an interpolated shape reconstruction and initial and final reconstructions, divided by the reconstruction error between initial and final reconstructions. This energy is computed across 10 interpolations between two given shapes. We computed the evolution energy for 20 random pairs of examples from the test set given each modality and dataset to obtain error bars. In the case of the cellPACK dataset, we computed the evolution energy for the entire test set comprising 117 examples.

Expressivity: *Rotation invariance error* is computed as the norm of the Euclidean distance between the embedding of a test set shape and its rotated version, divided by the sum of the norms of the two embeddings. The metric aims to quantify how much the embeddings change in representation space with rotation, while accounting for the size of the embedding space itself via

$$error = \frac{|z_\theta - z_0|}{||z_\theta| + |z_0||}, \text{ where } \theta \text{ is the rotation angle}$$

By dividing by the sum of the norms of the two embeddings, we establish their size relative to the origin. We computed this metric for four 90-degree rotations of the input in the XY plane. This is then averaged across many test set examples. *Average interpolation distance* is computed as the distance to the closest real example in representation space for interpolations between random test set examples (Extended Data Fig. 1b). We did this for 10 interpolations between randomly sampled pairs of test set examples and reported the average across pairs and interpolations. We computed the interpolation distance for 20 random pairs of examples from the test set given each modality and dataset to obtain error bars, like the evolution energy calculation. *Compactness* is an intrinsic dimensionality measure calculated using the Levina-Bickel score[1]. This score uses maximum likelihood estimation and a Poisson process approximation to examine the local neighborhood around each point and compute distances. All metrics were z-scored across models for polar plot visualization. The sign of all metrics except for classification and regression scores were flipped to ensure that a higher value indicates better performance. *Classification* and *regression scores* are the respective cross-validated test set accuracies and R^2 values for selected features. More details on classification and regression calculations can be found in section 1.2.

1.1 Reconstruction error

Punctate structures: We used the Chamfer loss to compute the reconstruction error of image- and point cloud-based models. Because this loss is normally applied to point clouds, we convert inputs and respective reconstructions from image models into point clouds by sampling points using the exponential sampling function described in section 3.1 We report test set reconstruction losses for each dataset. This comprised 234 samples for the cellPACK dataset, 122 samples for the PCNA dataset, and 7,620 samples for the punctate structure dataset.

Polymorphic structures: We used the Jaccard similarity score on binary masks to compute the reconstruction error of image- (segmentation and SDF) and point cloud-based models. For segmentation models, we used Otsu thresholding to binarize each reconstruction and extracted a mesh using marching cubes. Next, we upscaled the mesh and voxelized it into a 3D binary mask. To generate a binary mask for SDF images used as input for SDF models and their reconstructions, we first used the marching cubes algorithm to extract a zero-level set mesh. Then we upscaled the mesh and voxelized it to create a 3D binary mask. For SDF point cloud models, we used vertices of a 32x32x32 grid as XYZ query points to perform inference along with ground truth surface point clouds to obtain SDF reconstruction images. We then used the marching cubes algorithm to extract a zero-level set mesh from each SDF image, upscaled the mesh, and voxelized it into a 3D binary mask. We report test set reconstruction losses for each dataset. This comprised 1,773 samples for the nucleoli (GC) dataset, and 5,706 samples for the polymorphic structures dataset.

1.2 Classification and Regression Scores

We normalized embeddings using *StandardScaler* from scikit-learn. For classification accuracies, we used a logistic regression classifier with class weights and reported test set classification accuracies with stratified k-fold cross-validation with five splits. For regression scores, we used a linear regression model and reported test set R^2 with repeated k-fold cross validation with five splits and 20 repeats.

Classification and regression targets were designed appropriately. For the cellPACK synthetic dataset, we reported cross-validated accuracy for classifying the six packing rules. For the DNA replication foci dataset, we reported cross-validated top 2 classification accuracy for 8 interphase cell cycle stages and cross-validated accuracy for manually annotated outlier labels including dead cells and cells with no fluorescence. For punctate structures from the WTC-11 hiPSC Single-Cell Image Dataset v1, we reported cross-validated structure classification accuracy for 7 structures and cross-validated classification accuracy for cell cycle stages including interphase, prophase, early prometaphase,

prometaphase/metaphase and anaphase/telophase. For the nucleoli (GC) dataset, we reported cross-validated classification accuracy of thresholded number of pieces of nucleoli present in the segmentation, including 1, 2, 3, 4 and >=5 piece nucleoli. For this dataset, we also reported an average test set R^2 for regression of mean and standard deviation of piece volume and area, and an average test set R^2 for regression of mean and standard deviation of centroid-centroid distances between pieces. For the polymorphic structure dataset including nucleoli (GC and DFC), lysosomes and Golgi, we reported cross validated classification accuracy for structures, and regression scores for volume and area of pieces, and distance between pieces similar to the nucleoli (GC) dataset.

2. Model Background

Deep learning for feature extraction has been shown to be powerful in the context of cell biology, in particular, for analyzing images in 2D[2–4]. Despite its success, feature interpretability and generalizability to unseen image data continues to be a major challenge[5,6]. To alleviate some of these problems, it has been shown that imposing additional constraints corresponding to prior biological knowledge to models helps to reduce the space of admissible solutions and improve the likelihood that the learned features can be useful for scientific discovery[7].

One approach for imposing extra constraints informed by prior knowledge is the integration of known symmetries into the learning process. This has been the guiding principle for the field of geometric deep learning, a subset of machine learning that aims to exploit geometric principles and avoid the curse of dimensionality associated with learning generic functions in high-dimensional spaces[8]. While identifying symmetries in real-life datasets is not always straightforward, enforcing that learned representations are invariant or equivariant under simple Euclidean geometric transformations of the input data has been shown to improve data efficiency and generalization in fields like protein structure prediction[9], medical image analysis[10], and cell biology[11]. The distinction between invariant and equivariant representations is of note. While the first type of representation stays the same when the input data is subject to some transformation, the second changes predictably and equivalently. Rotation is a natural choice of geometrical transformation to extract features that do not depend on the object’s orientation.

3. Model training

3.1. Train, validation and test splits

We used a train/valid/test split ratio of 70%, 15%, 15% respectively across all datasets. For the expanded polymorphic dataset, we stratified the split using the structure class. For the perturbed nucleoli (GC) dataset, we stratified the split by drug.

3.2 Jitter augmentation

We trained the rotation invariant point cloud models with an augmented version of the DNA replication foci dataset (N=2,420) and the synthetic dataset from cellPACK (N=254) because of the small size of these datasets. This augmentation was done by adding jitter to each input point cloud (with 2,048 points) 10 times during training. The jitter was added via Gaussian noise with a standard deviation of 0.1. The noise was clipped at a value of 0.2. The typical range of XYZ coordinates was -10 to 10. Adding augmentations helped improve the quality of reconstructions (Supplementary Fig. 2g) for the planar and radial rules.

4. Validating orientation invariance of the rotation invariant models

To test the model implementations, we evaluated all models using different orientations of input shapes for the cellPACK synthetic dataset (Supplementary Fig. 1). First, we observed that rotations of the same input image and point cloud (first row in Supplementary Fig. 1) led to reconstructions with different orientations by the classical models (second row in Supplementary Fig. 1), highlighting the fact that these models are not rotation invariant. Next, we confirmed that the rotation invariant model reconstructions were indistinguishable across different rotations of the input (third row in Supplementary Fig. 1). Thus, the reconstructions and therefore the representations that they were created from, are rotation invariant. These rotation invariant reconstructions were then re-oriented to the pose of the input data (last row in Supplementary Fig. 1) using the learned rotation matrix for visual validation.

By visualizing rotation invariant reconstructions, we can focus on subtle differences in spatial patterns. For example, in the case of the cellPACK synthetic dataset, the rotation invariant reconstructions highlight the subtle differences in spatial distribution between 0- and 45-degrees orientation. Specifically, the reconstructions at 2σ for Planar 0 (Fig. 2c, upper left) are symmetric about the ZY plane, whereas the reconstructions at 2σ for Planar 45 (Fig. 2c, middle left) are asymmetric about the ZY plane (compare black arrows in Fig. 2c). This is because packing an elongated nucleus using the Planar 0 rule leads to a

symmetric gradient in the distribution of points, whereas the Planar 45 rule leads to more points being packed along the top and bottom of the nucleus compared to its sides (compare black arrows in Fig. 2a).

5. Data analysis

5.1 Principal component analysis (PCA)

We fit PCA to the learned rotation invariant representations using *sklearn.decomposition.PCA* with "auto" SVD solver and the number of components equal to the size of the embedding space. We then performed inverse PCA on different map points (-2σ, 0, 2σ) for different principal components (PCs). We used the inverse PCA reconstruction as input to the decoder of the representation learning model to visualize the reconstructed image/point cloud.

5.2 Archetype analysis

Archetype analysis approximates the convex hull of data points by identifying extremal points[12]. We used an implementation of the Frank-Wolfe algorithm for archetype analysis[12]. The number of archetypes for each dataset was chosen based on an expected number of clusters in each application. When prior knowledge is not available, the number of archetypes could be selected using heuristics such as visualization/interpretation, or via bootstrapping different numbers of archetypes and selecting the number that minimized the reconstruction error or maximized the fitness score.

5.3 Nuclear volume binning for nucleoli (GC) and DNA replication foci dataset

We clipped the nuclear volume distribution to be within the 2.5% and 97.5% range for both DNA replication foci and nucleolar GC datasets. Next, we binned the data into equal sized bins of 121 μm^3. The bins were <390 μm^3, 390-533 μm^3, 533-676 μm^3, 676-818 μm^3, and >818 μm^3.

5.4 Classification of number of pieces for nucleoli (GC)

We trained a logistic regression classifier with class weights to predict the number of nucleolar pieces (1, 2, 3, 4, and >4 pieces) for each bin of nuclear volume. We evaluated the classifier by using a five-fold stratified cross-validation.

5.5 Linear Discriminant Analysis (LDA)

To perform the LDA analysis on the nucleoli drug perturbation dataset, we first fit a PCA model to learned rotation invariant point cloud representations of the whole dataset. We used N=20 principal

components which retained 80% of the original variance. Next. we analyzed each control-drug pair separately by fitting a linear discriminant analysis (LDA) model. LDA was computed using *sklearn.discriminant_analysis.LinearDiscriminantAnalysis* with "svd" solver. We interpreted the main discriminant axis by sampling the closest real cells along the LDA line using computing Euclidean distances in the 20-dimensional PC space. We traversed the LDA line starting from the control population and dropped already retrieved samples along the way to avoid repeats.

We also fit LDA to random subsets of control sampled stratified by different plates. The control dataset comprised 8 unique plates and we randomly subset this into two sets of four. Next, we found the main LDA axis that separates the two subsets, and we traversed this axis starting from one subset towards the second subset. We sampled the closest real cell while dropping already retrieved samples along the way to avoid repeats.

6. Adapting the architecture for polymorphic structures

A few modifications had to be made to the autoencoder architecture to adapt it for polymorphic structures. First, while the encoder was left unchanged, its input point clouds are now sampled from segmented images instead of raw data, specifically from the surfaces of each of the multi-piece substructures. Next, a second point cloud is sampled from the 3D segmentation volume, and each point is assigned to its local SDF value. We replaced the point cloud decoder with an implicit decoder, which takes the vector latent representation and spatial coordinates of each point's location in the second point cloud as input, to reconstruct SDF values at each spatial location (Fig. 1c). Thus, both the encoder and decoder represent the 3D shape in different formats, allowing the model to capture complex shape information using SDFs, while remaining less sensitive to orientation changes.

More specifically, we reconstructed the rotation equivariant representation computed using vector neurons via an inner product decoder[13] to get signed distance function values at query points. These query points correspond to the points of the second point cloud sampled from the SDF volume as described in section 3.2. The model is optimized using an L1 loss on the SDF values. We took the norm of the embeddings after training to compute a rotation invariant representation. This relaxes the generative nature of the model as we are no longer able to obtain a rotation invariant reconstruction. This trades off the ability to generate rotation-invariant predictions with reconstruction quality, deemed necessary for the more complex shapes of the polymorphic structures. Instead, we visualize the closest real examples using Euclidean distances to different map points in the representation space (Fig. 5, 6).

7. Representations enable profiling on multiple polymorphic structures

We extended the application of our framework to profile multi-piece shape variation on the granular component (GC) of nucleoli (N=11,814) and three other polymorphic structures. To do this, we analyzed a subset of polymorphic structures from the WTC-11 hiPSC Single-Cell Image Dataset v1 (*Section 1.2 of Methods*). This subset comprised the dense fibrillar component (DFC) of nucleoli (N=9,923), lysosomes (N=10,114), and Golgi (N=6,175). Representative images of each structure are shown in Extended Data Figure 6a. We applied the SDF point cloud rotation invariant representation learning framework to this dataset of four different polymorphic structures, and once again benchmarked it against classical and rotation invariant segmentation-based and SDF-based image models (Extended Data Fig. 4). We focused here on learning scale invariant representations by scaling all intracellular structures on a cell-by-cell basis (*Section 2.2.2 of Methods,* Extended Data Fig. 4).

We again observed that the rotation invariant point cloud representations continued to display the lowest rotation invariance errors of any model tested (Extended Data Fig. 6b). We found that all rotation invariant models were more compact than their classical counterparts. We also found that both rotation invariant image models were worse at reconstruction and evolution energy than their classical counterparts. To evaluate the practical applicability of these models, we examined their performance on an intracellular structure classification task. All models achieved comparable accuracy in distinguishing between different intracellular structures ("Structure classification" in Extended Data Fig. 6b). This suggests that the morphology of these structures is distinct enough to be easily discerned using unsupervised learned representations, despite all of these being referred to as "polymorphic".

We performed PCA on rotation invariant point cloud representations learned for each of the four intracellular structures independently and visualized the first two principal components (Extended Data Fig. 6c-d). We identified features like overall elongation and number of pieces as primary sources of variation for both components of nucleoli, which are known to be nested via liquid-liquid phase separation[30]. In the case of lysosomes, we observed PC1 and PC2 to be related to aspects of cell shape. This was further confirmed by computing Pearson correlation between PC1 (explained variance ~15%) and cell elongation (r=-0.42), and PC2 (explained variance ~8%) and cell height (r=0.28). PC1 also displayed strong correlation with the average distance between lysosome pieces (r=-0.45 for PC1), suggesting that the first principal component also captures information about lysosome spatial clustering.

In the case of Golgi, the first two principal components (explained variance ~13% for PC1 and 9% for PC2) appeared to capture aspects of elongation and fragmentation. This was corroborated by computing correlations with structure elongation (r=-0.61 for PC1), and the average distance between Golgi pieces (r=-0.55 for PC1 and r=0.27 for PC2).

Next, we performed an archetype analysis with four archetypes given the representations of all structures and visualized them using a PaCMAP[14] projection (Extended Data Fig 6e). We colored the projection by the intracellular structure label and observed one well-separated cluster corresponding to nucleoli (GC), and three heterogeneous clusters corresponding to nucleoli (DFC), Golgi, and lysosomes (Extended Data Fig. 6e). We then projected the four archetypes onto this PaCMAP space and observed that each archetype localized well to each structure cluster, with archetypes 2, 3, and 4 localizing to the heterogeneous clusters. Because some clusters are very heterogeneous, we sampled the five closest real cells to each archetype (Extended Data Fig. 6f), instead of only one. We found that archetypes 1 and 2 were homogenous in the retrieved structure as all five closest examples corresponded to nucleoli (GC) and nucleoli (DFC) respectively. We observed some variability related to size and number of pieces amongst the sampled shapes, which can be attributed to the correlation between the PaCMAP dimensions and volume (r=0.57 for PaCMAP 1 and r=0.46 for PaCMAP 2), area (r=54 for PaCMAP 1 and r=0.45 for PaCMAP 2), and distance between pieces (r=0.48 for PaCMAP 1 and r=0.42 for PaCMAP 2). On the other hand, we observed that archetypes 3 and 4 were heterogeneous in the retrieved structure as some of the five closest examples did not correspond to the same structure. In the case of archetype 3, the fifth closest example was a nucleolus (GC) example, whereas in the case of archetype 4, the first and third closest examples were nucleoli (GC) and Golgi. Despite this, most of the closest examples belonged to the relevant structure, highlighting that each archetype represents the archetypical morphology corresponding to each structure.

Overall, we observed that no model performed well across all metrics, suggesting that application-appropriate model selection is key for achieving optimal results. The rotation invariant point cloud SDF model was biased towards good reconstruction scores, low rotation invariance errors, and performant regression and classification scores. In this case, we prioritized a combination of these metrics to pick the best model. However, if the goal is to learn a model that can regress biologically relevant information, then the rotation invariant image SDF model would be most appropriate.

8. Cellprofiler pipeline

We implemented a 3D feature extraction pipeline using Cellprofiler to use as a baseline for the perturbation detection task with the nucleolar perturbation dataset (Fig. 7). We selected the "Process as 3D" flag under the NamesAndTypes module with a relative pixel spacing of 0.108 in X, 0.108 in Y, and 0.29 in Z. We used four modules to process the 3D nucleolus (GC) segmentation images - ConvertImageToObjects, MeasureObjectSizeShape, MeasureObjectNeighbors, ExportToSpreadsheet. For the ConvertImageToObjects module, we converted the image to a boolean image and used background label = 0 and connectivity = 3. For the MeasureObjectSizeShape module, we calculated only Zernike features, as advanced feature calculation for the solidity measure resulted in Qhull errors related to forming a simplex. For the MeasureObjectNeighbors module, we used the Adjacent method to determine neighbors and did not discard objects touching the border. For the ExportToSpreadsheet module, we calculated the per-image mean, median, and standard-deviation values. This resulted in 81 features that were used for the q-value statistical analysis.

9. Discussion (continued)

We first tested our framework by recovering the rules used to create a synthetic dataset of punctate structures using cellPACK in a fully unsupervised way. We then recovered expected morphological patterns for different intracellular structures using unsupervised methods, such as archetype analysis and PCA, and by identifying biologically interpretable localization patterns for structures, like DNA replication foci and centrioles. Interestingly, the good performance achieved by different models in classifying cell cycle stages using PCNA suggests that manual annotation of this information can be replaced or assisted by unsupervised representation learning.

We further showed how the framework can be extended to polymorphic structures and characterized the full complexity of nucleolar shape variation as an example. In all cases, we observed distinct clustering in PCA space according to known biological phenotypes (Extended Data Figure 8). When applied to a larger dataset of polymorphic structures, the unsupervised representation learning revealed interesting spatial organization aspects of these structures. For example, Golgi fragmentation captured by the first two principal components of rotation invariant representations is often described as an important morphological feature under both normal and physiological conditions and has connections with different signaling pathways[15].

Finally, we evaluated the utility of our approach on phenotypic profiling of a nucleoli perturbed image dataset and demonstrated the interpretability of the learned representations. Using these representations to compute the mean morphology as well as the variability around the mean may reveal new phenotypes, which can be important in clinical settings. Classification metrics, such as uncertainty measures indicating multi-class membership, could also help quantify this variability. It remains unclear why some conditions associated with unhealthy cells were not picked up by any of the models. A larger drug screening single-cell image dataset is necessary to comprehensively validate our findings and evaluate the robustness of our approach.

Our study reveals that despite the success of point clouds to characterize punctate signal distribution in space, they are not sufficient to describe the shape of polymorphic organelles, such as nucleoli. This is exacerbated by the fact that rotation invariant image models reconstruct worse than classical image models across different datasets (Supplementary Figs. 2-7), despite learning more expressive representations in many cases (Fig. 3, 5, and Extended Data Fig. 6). This is because rotation invariance can reduce the functional form of the neural network, thereby reducing the space of admissible solutions, leading to worse reconstructions[16]. This motivated us to combine SDFs and point clouds for structures where shape is biologically important. By doing so, we observed a trade-off when using the rotation invariant point cloud SDF model where the model produced good reconstructions and learned expressive representations but was computationally inefficient compared to image models. This suggests that this model can be challenging to scale to larger datasets, as it would require expensive computational resources. Another potential pitfall that users should be cautious of is that the sampling function for point clouds from raw images can be sensitive to the signal to noise ratio, thus requiring validation for each new dataset.

In this work, we focused the experiments on 3D confocal images of intracellular structures in the hiPS cell, but our framework is general and can be applied to other cell types and imaging modalities. For example, single-molecule localization microscopy (SMLM) naturally generates large 3D point cloud data for single molecule interaction at the 10-20 nm resolution range[17]. Similarly, imaging-based spatial transcriptomics methods that measure a small subset of genes using single-molecule fluorescence in-situ hybridization (smFISH) technologies typically generate point locations of RNA localization[18], and antibody-based immunofluorescence imaging methods generate protein localization maps in 3D systems such as organoids[19,20]. These datasets are often quantified using point cloud clustering analysis and could benefit from unsupervised representation learning frameworks such as the one proposed here.

Many current techniques for analyzing single-molecule localization microscopy operate in two dimensions, forcing researchers to project their 3D data into 2D for analysis purposes[21,22]. This trend is prevalent in cell biology and medicine, where numerous publicly accessible datasets are predominantly in 2D[23–25]. The development of 3D analysis frameworks combined with availability of 3D data, can mitigate barriers to conducting comprehensive 3D analyses across diverse imaging modalities. This, in turn, reduces the risk of artifacts and allows analysis to be centered on the native data formats.

Previous studies have introduced unsupervised representation learning approaches for cell images using autoencoders with geometric deep learning[11,26]. Our work complements these approaches in three ways: first, by incorporating the notion of orientation invariance into our intracellular structure morphology-dependent framework for representation learning; second, by providing a systematic multi-task benchmark to evaluate the utility of each model that goes well beyond traditionally assessed reconstruction quality; third, by focusing our analysis on 3D multi-piece intracellular structures with complex morphology and spatial distribution. Our approach was tested across datasets of different sizes commonly obtained in typical single cell imaging studies, ranging from a few hundred to tens of thousands of samples, thus providing a guideline for the quality of unsupervised representations in different cases. For instance, we observed poor image-based reconstructions on small datasets like the DNA replication foci dataset (N=2,420). This observation might be closely related to our deliberate choice of using vanilla autoencoders throughout our analysis, and many modifications of autoencoders have been shown to improve different aspects of reconstruction[27,28], disentanglement[11,29,30], causal inference[31,32], and dynamics[33,34]. For example, we observed improved classical image reconstructions when using a masked autoencoder with a vision transformer backbone (MAE-ViT, Supplementary Figs. 3-4). As a trade-off, this model learns a high-dimensional latent space (4,096 patches x 256 dimensions). Exploring ways to extract interpretable information from this high-dimensional space is an exciting future direction of work[35].

References

1. Levina, E. & Bickel, P. Maximum Likelihood Estimation of Intrinsic Dimension. in *Advances in Neural Information Processing Systems* vol. 17 (MIT Press, 2004).
2. Ulicna, K., Kelkar, M., Soelistyo, C. J., Charras, G. T. & Lowe, A. R. Learning dynamic image representations for self-supervised cell cycle annotation. 2023.05.30.542796 Preprint at https://doi.org/10.1101/2023.05.30.542796 (2023).

3. Soelistyo, C. J., Vallardi, G., Charras, G. & Lowe, A. R. Learning biophysical determinants of cell fate with deep neural networks. *Nat. Mach. Intell.* **4**, 636–644 (2022).
4. Ternes, L. *et al.* A multi-encoder variational autoencoder controls multiple transformational features in single-cell image analysis. *Commun. Biol.* **5**, 1–10 (2022).
5. Locatello, F. *et al.* Challenging Common Assumptions in the Unsupervised Learning of Disentangled Representations. in *Proceedings of the 36th International Conference on Machine Learning* 4114–4124 (PMLR, 2019).
6. Rotem, O. *et al.* Visual interpretability of image-based classification models by generative latent space disentanglement applied to in vitro fertilization. 2023.11.15.566968 Preprint at https://doi.org/10.1101/2023.11.15.566968 (2023).
7. Soelistyo, C. J. & Lowe, A. R. Discovering interpretable models of scientific image data with deep learning. Preprint at https://doi.org/10.48550/arXiv.2402.03115 (2024).
8. Bronstein, M. M., Bruna, J., LeCun, Y., Szlam, A. & Vandergheynst, P. Geometric Deep Learning: Going beyond Euclidean data. *IEEE Signal Process. Mag.* **34**, 18–42 (2017).
9. Jumper, J. *et al.* Highly accurate protein structure prediction with AlphaFold. *Nature* **596**, 583–589 (2021).
10. Bekkers, E. J. *et al.* Roto-Translation Covariant Convolutional Networks for Medical Image Analysis. in *Medical Image Computing and Computer Assisted Intervention – MICCAI 2018* (eds. Frangi, A. F., Schnabel, J. A., Davatzikos, C., Alberola-López, C. & Fichtinger, G.) 440–448 (Springer International Publishing, Cham, 2018). doi:10.1007/978-3-030-00928-1_50.
11. Burgess, J. *et al.* Orientation-invariant autoencoders learn robust representations for shape profiling of cells and organelles. *Nat. Commun.* **15**, 1022 (2024).
12. Bauckhage, C., Kersting, K., Hoppe, F. & Thurau, C. Archetypal analysis as an autoencoder. (2015).
13. Deng, C. *et al.* Vector Neurons: A General Framework for SO(3)-Equivariant Networks. in 12200–12209 (2021).

14. Wang, Y., Huang, H., Rudin, C. & Shaposhnik, Y. Understanding How Dimension Reduction Tools Work: An Empirical Approach to Deciphering t-SNE, UMAP, TriMap, and PaCMAP for Data Visualization. *J. Mach. Learn. Res.* **22**, 1–73 (2021).

15. Makhoul, C., Gosavi, P. & Gleeson, P. A. Golgi Dynamics: The Morphology of the Mammalian Golgi Apparatus in Health and Disease. *Front. Cell Dev. Biol.* **7**, (2019).

16. Winter, R., Bertolini, M., Le, T., Noe, F. & Clevert, D.-A. Unsupervised Learning of Group Invariant and Equivariant Representations. *Adv. Neural Inf. Process. Syst.* **35**, 31942–31956 (2022).

17. Khater, I. M., Nabi, I. R. & Hamarneh, G. A Review of Super-Resolution Single-Molecule Localization Microscopy Cluster Analysis and Quantification Methods. *Patterns* **1**, 100038 (2020).

18. Chen, K. H., Boettiger, A. N., Moffitt, J. R., Wang, S. & Zhuang, X. Spatially resolved, highly multiplexed RNA profiling in single cells. *Science* **348**, aaa6090 (2015).

19. Hayashi, M. *et al.* Robust induction of primordial germ cells of white rhinoceros on the brink of extinction. *Sci. Adv.* **8**, eabp9683 (2022).

20. Gut, G., Herrmann, M. D. & Pelkmans, L. Multiplexed protein maps link subcellular organization to cellular states. *Science* **361**, eaar7042 (2018).

21. Malkusch, S. *et al.* Coordinate-based colocalization analysis of single-molecule localization microscopy data. *Histochem. Cell Biol.* **137**, 1–10 (2012).

22. Haas, K. T., Lee, M., Esposito, A. & Venkitaraman, A. R. Single-molecule localization microscopy reveals molecular transactions during RAD51 filament assembly at cellular DNA damage sites. *Nucleic Acids Res.* **46**, 2398–2416 (2018).

23. Chandrasekaran, S. N. *et al.* Three million images and morphological profiles of cells treated with matched chemical and genetic perturbations. *Nat. Methods* 1–8 (2024) doi:10.1038/s41592-024-02241-6.

24. Simpson, A. L. *et al.* A large annotated medical image dataset for the development and evaluation of segmentation algorithms. Preprint at https://doi.org/10.48550/arXiv.1902.09063 (2019).

25. Pontén, F., Jirström, K. & Uhlen, M. The Human Protein Atlas—a tool for pathology. *J. Pathol.* **216**, 387–393 (2008).

26. Vries, M. D. *et al.* 3D single-cell shape analysis using geometric deep learning. 2022.06.17.496550 Preprint at https://doi.org/10.1101/2022.06.17.496550 (2023).

27. Razavi, A., van den Oord, A. & Vinyals, O. Generating Diverse High-Fidelity Images with VQ-VAE-2. in *Advances in Neural Information Processing Systems* vol. 32 (Curran Associates, Inc., 2019).

28. He, K. *et al.* Masked Autoencoders Are Scalable Vision Learners. in 16000–16009 (2022).

29. Kingma, D. P. & Welling, M. Auto-Encoding Variational Bayes. Preprint at https://doi.org/10.48550/arXiv.1312.6114 (2022).

30. Chen, R. T. Q., Li, X., Grosse, R. B. & Duvenaud, D. K. Isolating Sources of Disentanglement in Variational Autoencoders. in *Advances in Neural Information Processing Systems* vol. 31 (Curran Associates, Inc., 2018).

31. Sturma, N., Squires, C., Drton, M. & Uhler, C. Unpaired Multi-Domain Causal Representation Learning. *Adv. Neural Inf. Process. Syst.* **36**, 34465–34492 (2023).

32. Louizos, C. *et al.* Causal Effect Inference with Deep Latent-Variable Models. in *Advances in Neural Information Processing Systems* vol. 30 (Curran Associates, Inc., 2017).

33. Lusch, B., Kutz, J. N. & Brunton, S. L. Deep learning for universal linear embeddings of nonlinear dynamics. *Nat. Commun.* **9**, 4950 (2018).

34. Bakarji, J., Champion, K., Kutz, J. N. & Brunton, S. L. Discovering Governing Equations from Partial Measurements with Deep Delay Autoencoders. Preprint at https://doi.org/10.48550/arXiv.2201.05136 (2022).

35. Kraus, O. *et al.* Masked Autoencoders for Microscopy are Scalable Learners of Cellular Biology. in 11757–11768 (2024).

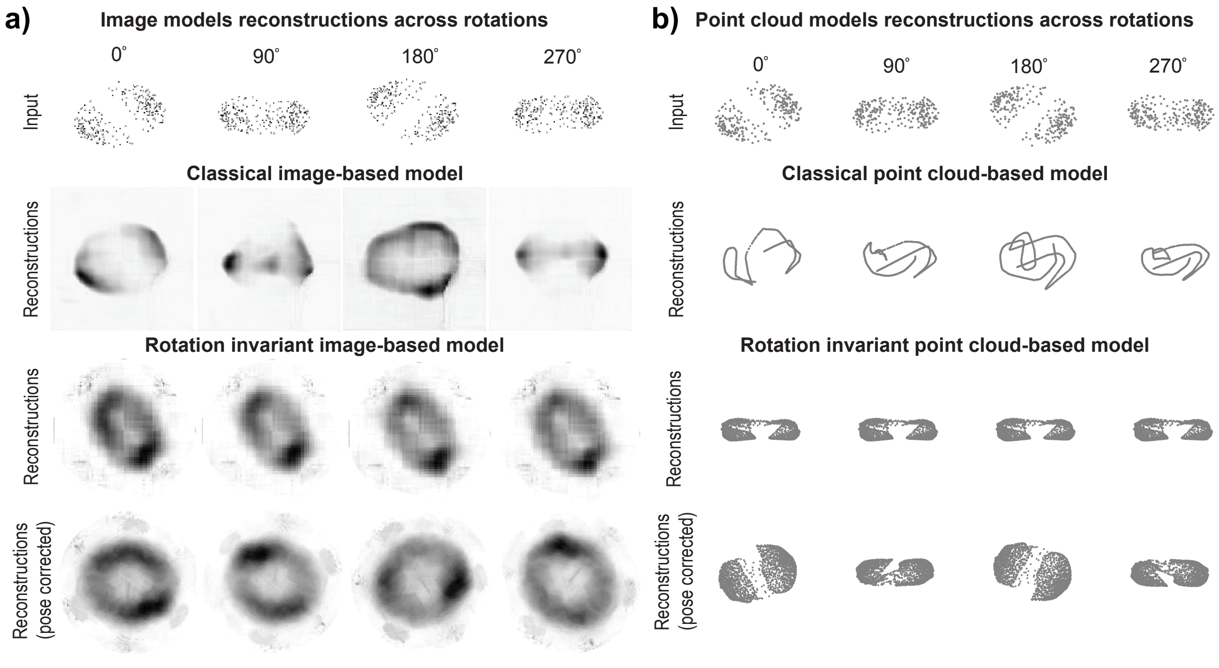

Figure S1 - Testing orientation invariance for image and point cloud models for the cellPACK synthetic dataset a) (Top row) Example image input for the planar 45 rule is rotated by four 90-degree rotations. (Second row) Reconstructions using the classical image model for each rotated input (Third row) Reconstructions using the rotation invariant image model for each rotated input. (Bottom row) Pose-corrected rotation invariant reconstructions using the rotation invariant image model for each rotated input. **b)** (Top row) Example point cloud input for the planar 45 rule is rotated by four 90-degree rotations. (Second row) Reconstructions using the classical point cloud model for each rotated input (Third row) Reconstructions using the rotation invariant point cloud model for each rotated input. (Bottom row) Pose-corrected rotation invariant reconstructions using the rotation invariant point cloud model for each rotated input. All reconstructions shown are max projections in Z.

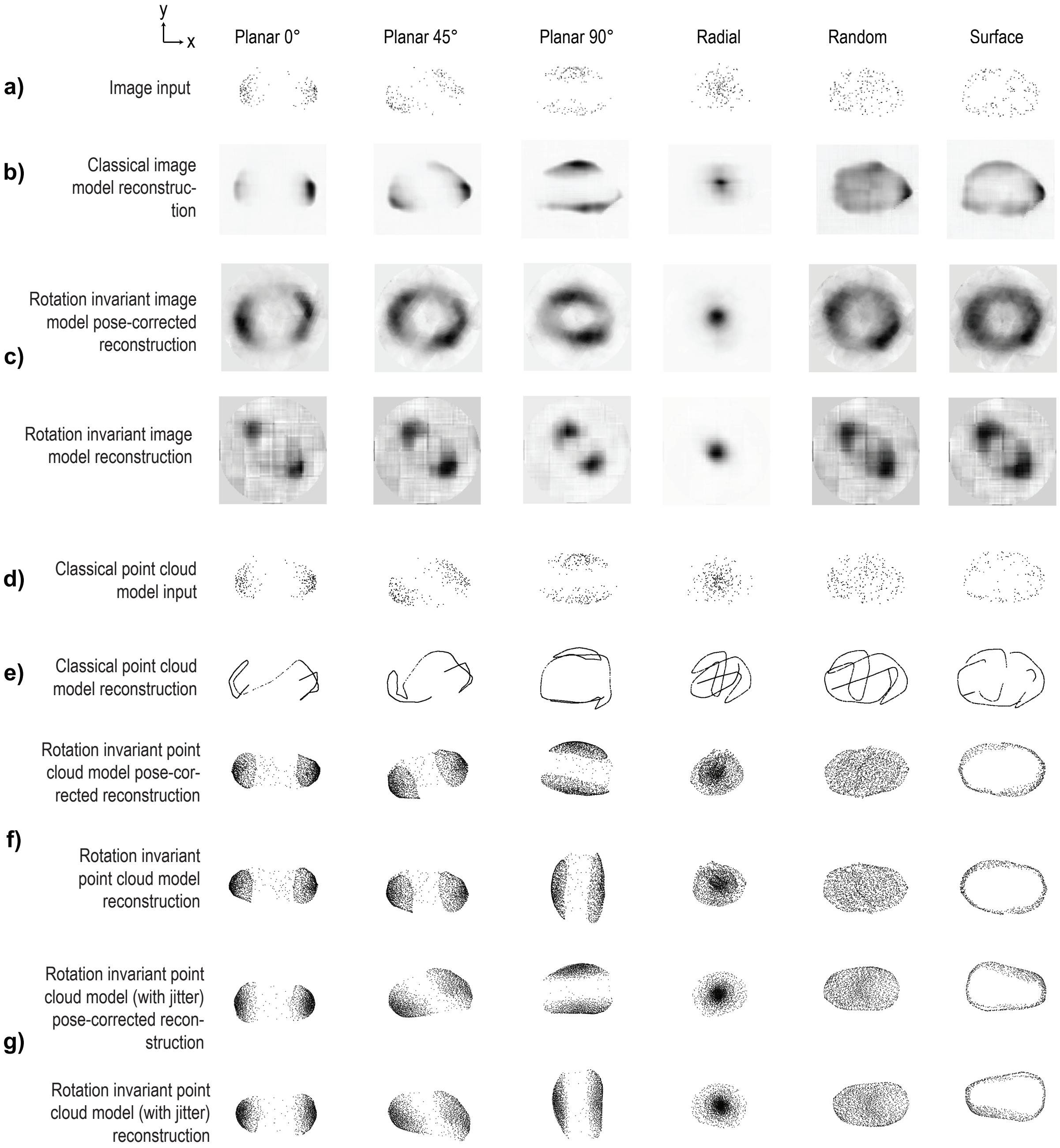

Figure S2 - Evaluation of test set model reconstructions for synthetic punctate structures generated using cellPACK. Test set center slice inputs (**a, d)** and reconstructions using **b)** classical image model, **c)** rotation invariant image model, **e)** classical point cloud model, **f)** rotation invariant point cloud model for each of the 6 packing rules, and g) rotation invariant point cloud model with jitter augmentations for each of the 6 packing rules. Both pose-corrected and rotation invariant reconstructions are shown for the rotation invariant models.

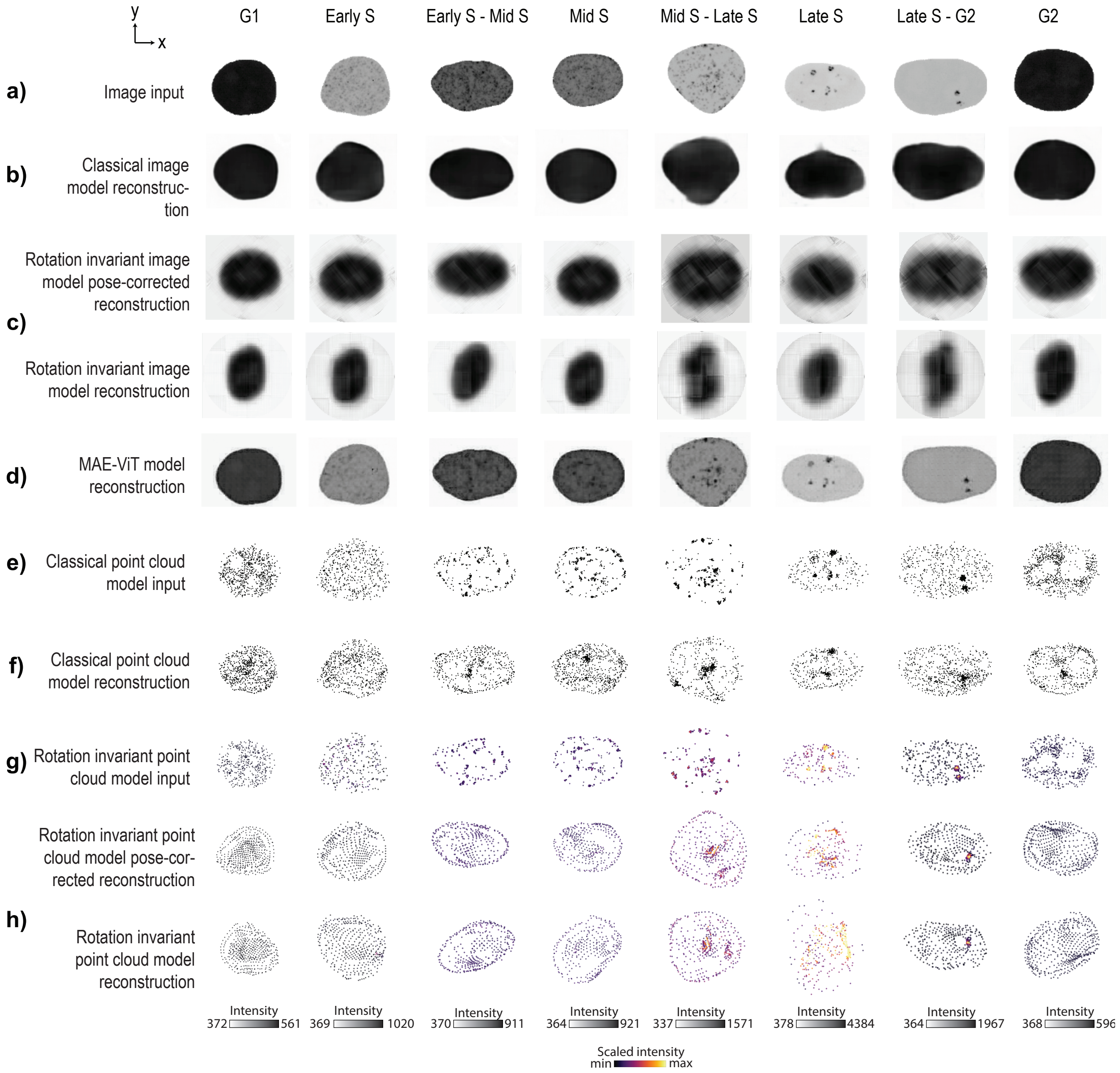

Figure S3 - Evaluation of test set model reconstructions for the DNA replication foci dataset. Test set center slice inputs (**a, e, g**) and reconstructions using **b)** classical image model, **c)** rotation invariant image model, **d)** an alternative classical image model via a masked autoencoder with a vision transformer as an encoder (MAE-ViT), **f)** classical point cloud model, and **h)** rotation invariant point cloud model for samples from each of the 8 cell cycle stages. Both pose-corrected and rotation invariant reconstructions are shown for the rotation invariant models.

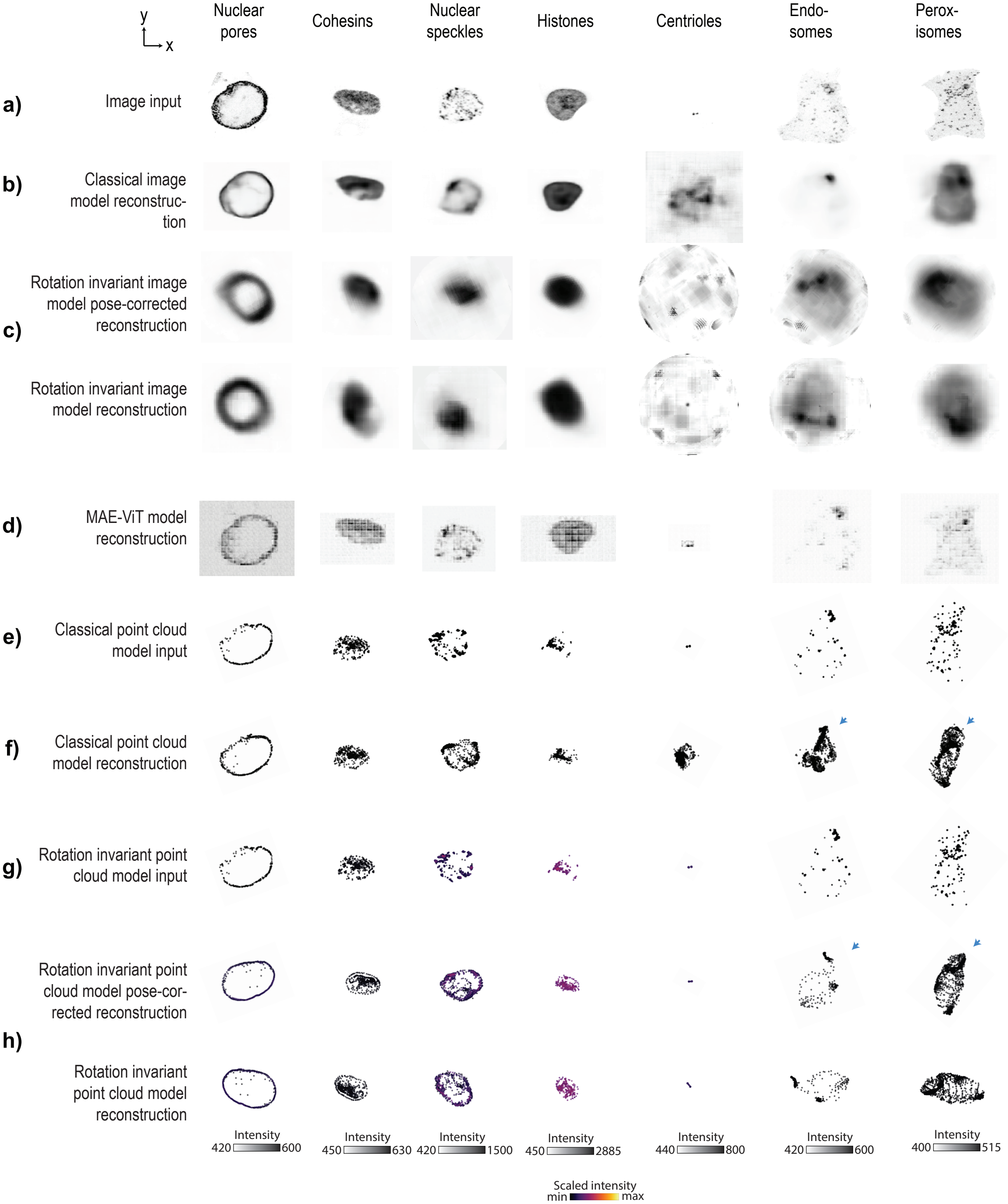

Figure S4 - Evaluation of test set model reconstructions for punctate structures from the WTC-11 hiPSC Single-Cell Image Dataset v1. Visualization of test set reconstructions for sampled histones (CellId 721646), nuclear pores (CellId 873680), cohesins (CellId 994027), nuclear speckles (CellId 490385), centrioles (CellId 451974), endosomes (CellId 811336), peroxisomes (CellId 835431). Shown are test set inputs (**a, e, g**) and reconstructions using **b)** classical image model, **c)** rotation invariant image

model, **d)** an alternative classical image model via a masked autoencoder with a vision transformer as an encoder (MAE-ViT), **f)** classical point cloud model, and **h)** rotation invariant point cloud model for each structure. Both pose-corrected and rotation invariant reconstructions are shown for the rotation invariant models. Reconstructions for nuclear pores, cohesins, and histones are center slices, whereas reconstructions for nuclear speckles, centrioles, endosomes, and peroxisomes are max projections. Spatial distribution artifacts in reconstructions for endosomes and peroxisomes are highlighted with blue arrows.

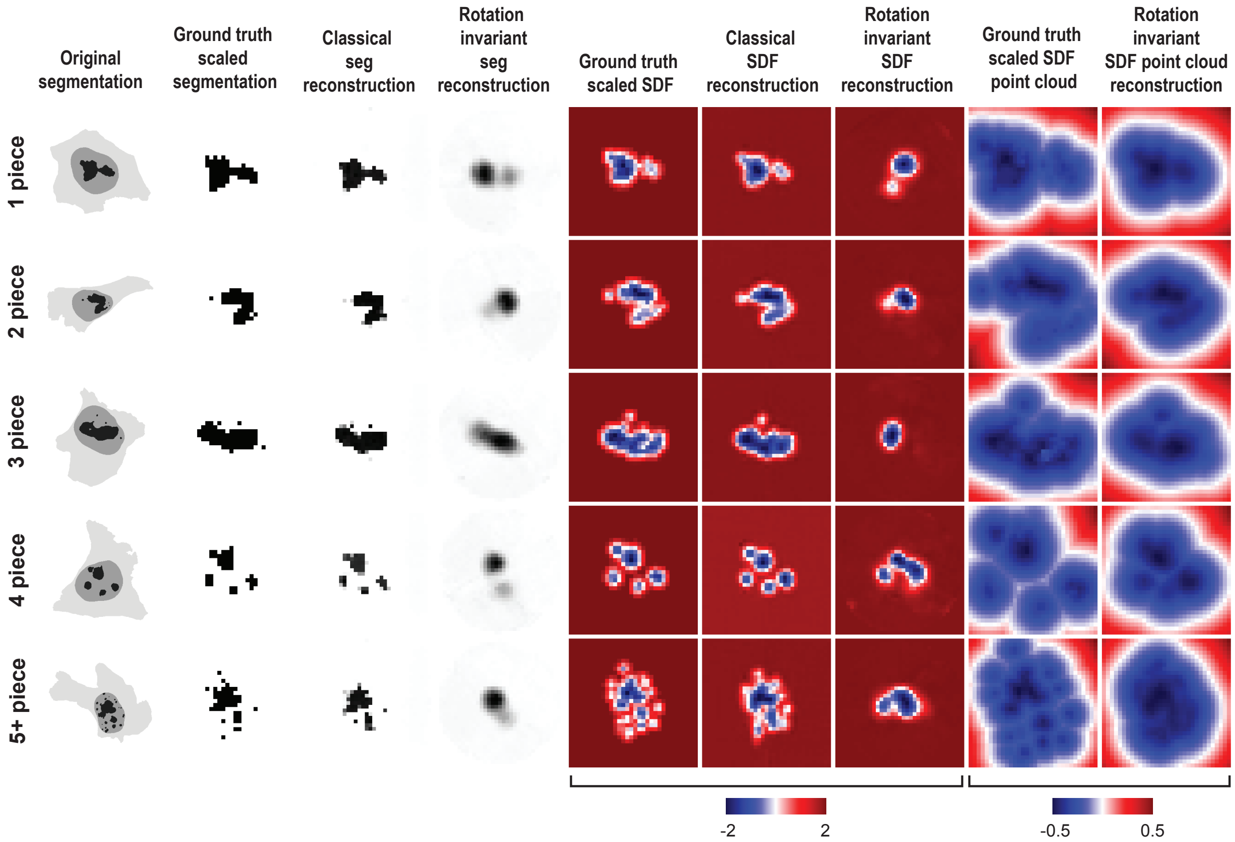

Figure S5 - Examples of model inputs and outputs for nucleolar GC dataset. Test set reconstructions across all models for sampled 1 piece (CellId 964798), 2 pieces (CellId 661110), 3 pieces (CellId 644401), 4 pieces (CellId 967887) and 5+ pieces (CellId 703621) examples. Max projections of original structure segmentations overlaid with nuclear and membrane segmentations are shown. Max projections are shown for segmentations, whereas middle slices are shown for SDFs.

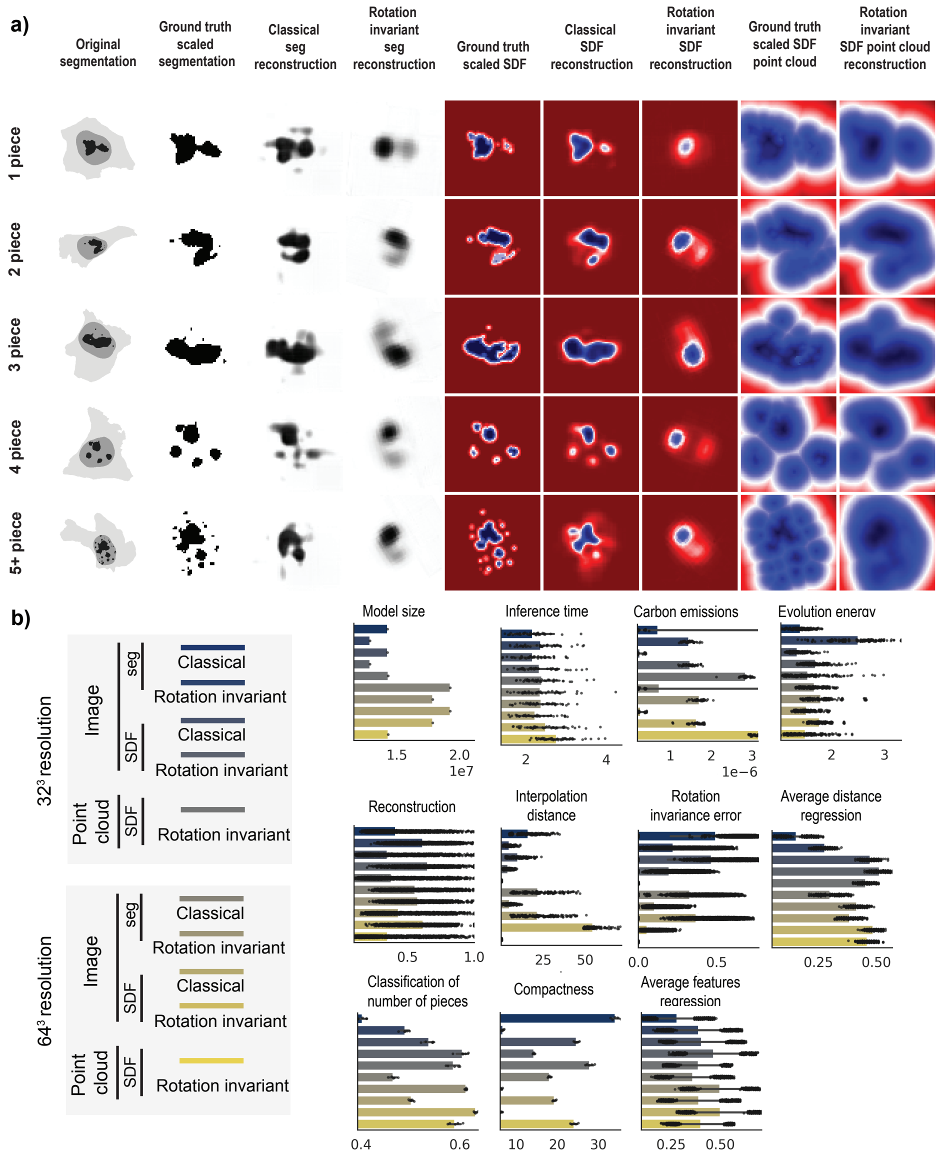

Figure S6 - Examples of model inputs and outputs for nucleolar GC dataset at 64^3 resolution. a) Test set reconstructions across all models for sampled 1-piece (CellId 964798), 2 pieces (CellId 661110), 3 pieces (CellId 644401), 4 pieces (CellId 967887) and 5+ pieces (CellId 703621) examples. All examples are scaled to 64^3 resolution. Max projections of original structure segmentations overlaid with nuclear and membrane segmentations are shown. Max projections are shown for segmentations, whereas middle slices are shown for SDFs. **b)** Bar plots showing benchmarking comparison between 32^3

resolution models and 64^3 resolution models across efficiency metrics (model size, inference time, emissions), generative metrics (reconstruction, evolution energy), representation expressivity metrics (compactness, classification of number of pieces, shape features regression, distance features regression, rotation invariance error, average interpolation distance). Error bars are standard deviations.

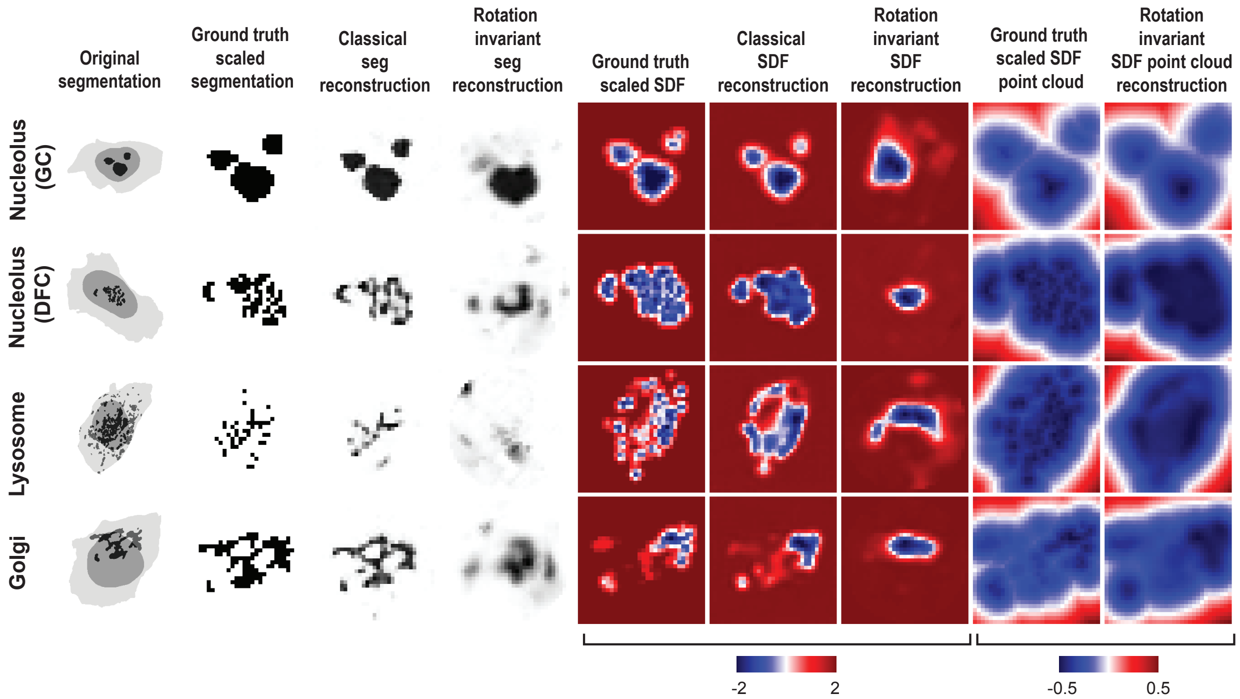

Figure S7 - Evaluation of model reconstructions for polymorphic structures from the WTC-11 hiPSC Single-Cell Image Dataset v1. Test set reconstructions across all models for sampled nucleoli (GC) (CellId 691110), nucleoli (DFC) (CellId 723687), lysosome (CellId 816468), and Golgi (CellId 800894) examples. Max projections of original structure segmentations overlaid with nuclear and membrane segmentations are shown. Max projections are shown for segmentations, whereas middle slices are shown for SDFs.

Drug	Manufacturer	ID	Concentration(s)
Actinomycin D	Selleckchem	S8964	0.5µg/mL
BIX 01294	Selleckchem	S8006	1µM
Bafilomycin A	Millipore Sigma	SML1661	0.1µM
Brefeldin	Selleckchem	S7046	5µM
Chloroquine	Medchemexpress	HY-17589A	40µM
H89	Selleckchem	S1582	10µM
Jasplakinolide	Millipore Sigma	420127	50nM
Latrunculin A1	Millipore Sigma	428026	0.1µM
Monensin A	Medchemexpress	HY-N0150	1.1µM
Nocodazole	Medchemexpress	HY-13520	0.1µM
Paclitaxel	Selleckchem	S1150	5µM
Rapamycin	Selleckchem	S1039	1µM
Roscovitine	Selleckchem	S1153	5µM and 10µM
Rotenone	Medchemexpress	HY-B1756	0.5µM
Staurosporine	Selleckchem	S1421	1µM
Torin-2	Selleckchem	S2817	1µM

Table 1 - List of drugs and concentrations used in the perturbed nucleolar GC dataset

Experimental models: Cell Lines	Source	Identifier
AICS-0014 cl. 6, nucleoli (DFC)	https://catalog.coriell.org/0/Sections/Search/Sample_Detail.aspx?Ref=AICS-0014&Product=iPSC	CVCL_JM17
AICS-0057 cl. 50, nucleoli (GC)	https://www.coriell.org/0/Sections/Search/Sample_Detail.aspx?Ref=AICS-0057-050&PgId=166	CVCL_VK85
AICS-0094 cl. 24, nuclear speckles	https://www.coriell.org/0/Sections/Search/Sample_Detail.aspx?Ref=AICS-0094-024&PgId=166	CVCL_YU30
AICS-0068 cl. 9, cohesins	https://www.coriell.org/0/Sections/Search/Sample_Detail.aspx?Ref=AICS-0068-009&PgId=166	CVCL_UK04
AICS-0061 cl. 36, histones	https://www.coriell.org/0/Sections/Search/Sample_Detail.aspx?Ref=AICS-0061-036&PgId=166	CVCL_UD17
AICS-0013 cl. 210, nuclear envelope	https://catalog.coriell.org/0/Sections/Search/Sample_Detail.aspx?Ref=AICS-0013&Product=iPSC	CVCL_IR32
AICS-0069 cl. 88, nuclear pores	https://www.coriell.org/0/Sections/Search/Sample_Detail.aspx?Ref=AICS-0069-088&PgId=166	CVCL_UD18
AICS-0033 cl. 115, peroxisomes	https://www.coriell.org/0/Sections/Search/Sample_Detail.aspx?Ref=AICS-0033-115&PgId=166	CVCL_VK79
AICS-0040 cl. 35, endosomes	https://www.coriell.org/0/Sections/Search/Sample_Detail.aspx?Ref=AICS-0040-035&PgId=166	CVCL_VK82
AICS-0022 cl. 37, lysosomes	https://catalog.coriell.org/0/Sections/Search/Sample_Detail.aspx?Ref=AICS-0022-037&Product=iPSC	CVCL_LK42

AICS-0025 cl. 44, Golgi	https://catalog.coriell.org/0/Sections/Search/Sample_Detail.aspx?Ref=AICS-0025-044&Product=iPSC	CVCL_LK43
AICS-0032 cl. 19, centrioles	https://catalog.coriell.org/0/Sections/Search/Sample_Detail.aspx?Ref=AICS-0032-019&Product=iPSC	CVCL_LK45
AICS-0088 cl. 83, DNA replication foci	https://www.coriell.org/0/Sections/Search/Sample_Detail.aspx?Ref=AICS-0088-083&PgId=166	CVCL_A8RT

Table 2 - List of all cell lines used in this study
