# Supplementary material for: Interpretable representation learning for 3D multi-piece intracellular structures using point clouds
Source: Nat Methods. 2025 Jul 3;22(7):1531–44. doi: 10.1038/s41592-025-02729-9 (PMC12240800; doi:10.1038/s41592-025-02729-9)
Supplement: Supplementary file 2 — Reporting Summary [file 41592_2025_2729_MOESM2_ESM.pdf]

nature portfolio

Corresponding author(s): Matheus Palhares Viana

Last updated by author(s): Apr 23, 2025

Reporting Summary

Nature Portfolio wishes to improve the reproducibility of the work that we publish. This form provides structure for consistency and transparency in reporting. For further information on Nature Portfolio policies, see our Editorial Policies and the Editorial Policy Checklist.

Statistics

For all statistical analyses, confirm that the following items are present in the figure legend, table legend, main text, or Methods section.

n/a	Confirmed	
☐	☒	The exact sample size (*n*) for each experimental group/condition, given as a discrete number and unit of measurement
☐	☒	A statement on whether measurements were taken from distinct samples or whether the same sample was measured repeatedly
☒	☐	The statistical test(s) used AND whether they are one- or two-sided<br>*Only common tests should be described solely by name; describe more complex techniques in the Methods section.*
☒	☐	A description of all covariates tested
☒	☐	A description of any assumptions or corrections, such as tests of normality and adjustment for multiple comparisons
☐	☒	A full description of the statistical parameters including central tendency (e.g. means) or other basic estimates (e.g. regression coefficient) AND variation (e.g. standard deviation) or associated estimates of uncertainty (e.g. confidence intervals)
☒	☐	For null hypothesis testing, the test statistic (e.g. *F*, *t*, *r*) with confidence intervals, effect sizes, degrees of freedom and *P* value noted<br>*Give P values as exact values whenever suitable.*
☒	☐	For Bayesian analysis, information on the choice of priors and Markov chain Monte Carlo settings
☒	☐	For hierarchical and complex designs, identification of the appropriate level for tests and full reporting of outcomes
☒	☐	Estimates of effect sizes (e.g. Cohen's *d*, Pearson's *r*), indicating how they were calculated

Our web collection on statistics for biologists contains articles on many of the points above.

Software and code

Policy information about availability of computer code

Data collection: cellPACK 1.0.8 for generating synthetic dataset and ZEN 2.3 software (blue edition; ZEISS) for imaging PCNA and drug perturbation dataset.

Data analysis: The versions of all Python packages used in this work are listed in the Github repositories (https://github.com/AllenCell/benchmarking_representations and https://github.com/AllenCellModeling/cyto-dl/tree/br_release). A full list is also provided here:

numpy==1.25.2
pdm-backend==2.4.3
pdm-pep517==1.1.4
setuptools==73.0.1
--find-links https://data.pyg.org/whl/torch-2.0.1+cu117.html
aicscytoparam==0.1.9
aicsimageio==4.11.0
aicsshparam==0.1.10
aiobotocore==2.4.2
aiohttp==3.9.3
aioitertools==0.11.0
aiosignal==1.3.1
alembic==1.13.1
anndata==0.9.2
annotated-types==0.6.0
annoy==1.17.3

```
ansicon==1.89.0; platform_system == "Windows"
antlr4-python3-runtime==4.9.3
anyio==4.3.0
appnope==0.1.4; platform_system == "Darwin"
arrow==1.3.0
asciitree==0.3.3
astropy==5.2.2
asttokens==2.4.1
async-timeout==4.0.3; python_version < "3.11"
attrs==23.2.0
autograd==1.6.2
autopage==0.5.2
backoff==2.2.1
beautifulsoup4==4.12.3
bfio==2.3.0
blessed==1.20.0
blinker==1.7.0
boto3==1.24.4
botocore==1.27.59
certifi==2024.2.2
cffi==1.17.1; implementation_name == "pypy"
charset-normalizer==3.3.2
click==8.1.7
cliff==4.6.0
cloudpickle==3.0.0
cmaes==0.10.0
cmake==3.28.3; platform_system == "Linux" and platform_machine == "x86_64"
cmd2==2.4.3
codecarbon==2.6.0
colorama==0.4.6
colorlog==6.8.2
comm==0.2.2
contourpy==1.1.1
copairs @ git+https://github.com/cytomining/copairs.git@880f22a551bd897896d148a0b07baa99d981c6a9
croniter==1.4.1
cycler==0.12.1
cyto-dl[equiv,pcloud,s3,spharm,torchserve] @ git+https://github.com/AllenCellModeling/cyto-
dl.git@a4a061d1808e0f94f906933642920142b581ba38
dask[array]==2023.5.0
dateutils==0.6.12
debugpy==1.8.7
decorator==5.1.1
deepdiff==6.7.1
deprecated==1.2.14
distributed==2023.5.0
docker==7.0.0
drjit==0.4.6
e3nn==0.5.1
edt==2.4.1
einops==0.7.0
elementpath==4.3.0
entrypoints==0.4
escnn==1.0.11
exceptiongroup==1.2.0; python_version < "3.11"
executing==2.1.0
fastapi==0.109.2
fasteners==0.19
filelock==3.13.1
fire==0.5.0
flask==3.0.2
fonttools==4.49.0
freetype-py==2.5.1
frozenlist==1.4.1
fsspec[http]==2023.3.0
fsspec[s3]==2023.3.0
future==1.0.0
geomloss==0.2.6
gitdb==4.0.11
gitpython==3.1.42
gorilla==0.4.0
greenlet==3.0.3; (platform_machine == "win32" or platform_machine == "WIN32" or platform_machine == "AMD64" or platform_machine ==
"amd64" or platform_machine == "x86_64" or platform_machine == "ppc64le" or platform_machine == "aarch64") and python_version >= "3"
gunicorn==21.2.0; platform_system != "Windows"
h11==0.14.0
h5py==3.10.0
huggingface-hub==0.17.3
```

```
humanize==4.9.0
hydra-colorlog==1.2.0
hydra-core==1.3.2
hydra-optuna-sweeper==1.2.0
idna==3.6
imagecodecs==2023.3.16
imageio==2.34.0
importlib-metadata==7.0.1
iniconfig==2.0.0
inquirer==3.1.4
ipykernel==6.29.5
ipython==8.27.0
itsdangerous==2.1.2
jedi==0.19.1
jinja2==3.1.3
jinxed==1.3.0; platform_system == "Windows"
jmespath==1.0.1
joblib==1.3.2
jupyter-client==8.6.3
jupyter-core==5.7.2
kaleido==0.2.1
kiwisolver==1.4.5
lazy-loader==0.3
lie-learn==0.0.1.post1
lightning==2.0.9.post0
lightning-cloud==0.5.64
lightning-utilities==0.10.1
lit==17.0.6; platform_system == "Linux" and platform_machine == "x86_64"
llvmlite==0.43.0
locket==1.0.0
lxml==4.9.4
mako==1.3.2
markdown==3.5.2
markdown-it-py==3.0.0
markupsafe==2.1.5
matplotlib==3.7.5
matplotlib-inline==0.1.7
mdurl==0.1.2
mesh-to-sdf==0.0.15
mitsuba==3.5.2
mlflow==2.10.2
monai-weekly==1.4.dev2407
mpmath==1.3.0
msgpack==1.0.7
multidict==6.0.5
natsort==8.4.0
nest-asyncio==1.6.0
networkx==3.1
nibabel==5.2.0
ninja==1.11.1.1
numba==0.60.0
numcodecs==0.12.1
numpy==1.25.2
nvidia-cublas-cu11==11.10.3.66; platform_system == "Linux" and platform_machine == "x86_64"
nvidia-cuda-cupti-cu11==11.7.101; platform_system == "Linux" and platform_machine == "x86_64"
nvidia-cuda-nvrtc-cu11==11.7.99; platform_system == "Linux" and platform_machine == "x86_64"
nvidia-cuda-runtime-cu11==11.7.99; platform_system == "Linux" and platform_machine == "x86_64"
nvidia-cudnn-cu11==8.5.0.96; platform_system == "Linux" and platform_machine == "x86_64"
nvidia-cufft-cu11==10.9.0.58; platform_system == "Linux" and platform_machine == "x86_64"
nvidia-curand-cu11==10.2.10.91; platform_system == "Linux" and platform_machine == "x86_64"
nvidia-cusolver-cu11==11.4.0.1; platform_system == "Linux" and platform_machine == "x86_64"
nvidia-cusparse-cu11==11.7.4.91; platform_system == "Linux" and platform_machine == "x86_64"
nvidia-nccl-cu11==2.14.3; platform_system == "Linux" and platform_machine == "x86_64"
nvidia-nvtx-cu11==11.7.91; platform_system == "Linux" and platform_machine == "x86_64"
ome-types==0.5.0
ome-zarr==0.8.3
omegaconf==2.3.0
opt-einsum==3.3.0
opt-einsum-fx==0.1.4
optuna==2.10.1
ordered-set==4.1.0
ostat==0.2
packaging==23.2
pacmap==0.7.3
pandas==1.5.3
parso==0.8.4
```

```
partd==1.4.1
patsy==0.5.6
pbr==6.0.0
pexpect==4.9.0; sys_platform != "win32" and sys_platform != "emscripten"
pillow==10.2.0
pip==24.3.1
platformdirs==4.2.0
plotly==5.24.1
pluggy==1.5.0
point-cloud-utils==0.30.4
pooch==1.8.1
prettytable==3.10.0
prometheus-client==0.21.0
prompt-toolkit==3.0.47
protobuf==3.20.1
psutil==5.9.8
ptyprocess==0.7.0; sys_platform != "win32" and sys_platform != "emscripten"
pure-eval==0.2.3
py-cpuinfo==9.0.0
py3nj==0.1.2
pyarrow==10.0.1
pycparser==2.22; implementation_name == "pypy"
pycytominer==1.2.0
pydantic==2.1.1
pydantic-compat==0.1.2
pydantic-core==2.4.0
pyerfa==2.0.0.3
pyglet==2.0.17
pygments==2.17.2
pyjwt==2.8.0
pymanopt==2.2.0
pymcubes==0.1.6
pyntcloud==0.3.1
pynvml==11.5.3
pyopengl==3.1.0
pyparsing==3.1.1
pyperclip==1.8.2
pyreadline3==3.5.4; sys_platform == "win32"
pyrender==0.1.45
pyrootutils==1.0.4
pyshtools==4.10.4
pytest==8.3.3
python-dateutil==2.8.2
python-dotenv==1.0.1
python-editor==1.0.4
python-multipart==0.0.9
pytorch-lightning==2.2.0.post0
pytz==2023.4
pyvista==0.44.1
pywavelets==1.4.1
pywin32==307; sys_platform == "win32"
pyyaml==6.0.1
pyzmq==26.2.0
querystring-parser==1.2.4
questionary==1.10.0
rapidfuzz==3.10.0
readchar==4.0.5
requests==2.31.0
resource-backed-dask-array==0.1.0
rich==13.7.0
s3fs==2023.3.0
s3transfer==0.6.2
safetensors==0.4.2
scikit-image==0.21.0
scikit-learn==1.3.2
scipy==1.9.3
scooby==0.10.0
seaborn==0.13.2
setuptools==73.0.1
shellingham==1.5.4
simpleitk==2.3.1
six==1.16.0
smmap==5.0.1
sniffio==1.3.0
sortedcontainers==2.4.0
soupsieve==2.5
```

```
sqlalchemy==1.4.54
sqlparse==0.4.4
stack-data==0.6.3
starlette==0.36.3
starsessions==1.3.0
statsmodels==0.14.3
stevedore==5.2.0
sympy==1.12
tblib==3.0.0
tenacity==9.0.0
termcolor==2.4.0
threadpoolctl==3.3.0
tifffile==2023.7.10
timm==0.9.16
tomli==2.1.0; python_version < "3.11"
toolz==0.12.1
torch==2.0.1
torch-geometric==2.5.0
torch-scatter==2.1.2
torchio==0.19.6
torchmetrics==1.3.1
torchserve==0.9.0
torchvision==0.15.2
tornado==6.4
tqdm==4.66.2
traitlets==5.14.1
trimesh==4.4.9
triton==2.0.0; platform_system == "Linux" and platform_machine == "x86_64"
typer[all]==0.9.0
types-python-dateutil==2.8.19.20240106
typing-extensions==4.9.0
universal-pathlib==0.2.1
urllib3==1.26.18
uvicorn==0.27.1
vn-transformer==0.1.0
vtk==9.3.0
waitress==2.1.2; platform_system == "Windows"
wcwidth==0.2.13
websocket-client==1.7.0
websockets==12.0
werkzeug==3.0.1
wheel==0.44.0
wrapt==1.16.0
xarray==2023.1.0
xmlschema==3.0.2
xsdata==24.2.1
yarl==1.9.4
zarr==2.16.1
zict==3.0.0
zipp==3.17.0
--find-links https://data.pyg.org/whl/torch-2.0.1+cu117.html
```

For manuscripts utilizing custom algorithms or software that are central to the research but not yet described in published literature, software must be made available to editors and reviewers. We strongly encourage code deposition in a community repository (e.g. GitHub). See the Nature Portfolio guidelines for submitting code & software for further information.

Data

Policy information about availability of data

All manuscripts must include a data availability statement. This statement should provide the following information, where applicable:

- Accession codes, unique identifiers, or web links for publicly available datasets
- A description of any restrictions on data availability
- For clinical datasets or third party data, please ensure that the statement adheres to our policy

The WTC-11 hiPSC single cell image dataset v1 analyzed in this study is available online at https://open.quiltdata.com/b/allencell/packages/aics/hipsc_single_cell_image_dataset. The DNA replication foci dataset analyzed in this study is available online at https://open.quiltdata.com/b/allencell/packages/aics/nuclear_project_dataset_4. The WTC-11 hiPSC nucleoli (NPM1) perturbation single cell image dataset analyzed in this study is available online at https://open.quiltdata.com/b/allencell/tree/aics/NPM1_single_cell_drug_perturbations/. The synthetic dataset of punctate structures generated using cellPACK and analyzed in this study is available online at https://open.quiltdata.com/b/allencell/tree/aics/morphology_appropriate_representation_learning/cellPACK_single_cell_punctate_structure/. The landing page of the GitHub repository associated with this manuscript (https://github.com/AllenCell/benchmarking_representations) has additional information for accessing and processing these datasets.

Research involving human participants, their data, or biological material

Policy information about studies with human participants or human data. See also policy information about sex, gender (identity/presentation), and sexual orientation and race, ethnicity and racism.

Reporting on sex and gender	N/A
Reporting on race, ethnicity, or other socially relevant groupings	N/A
Population characteristics	N/A
Recruitment	N/A
Ethics oversight	N/A

Note that full information on the approval of the study protocol must also be provided in the manuscript.

Field-specific reporting

Please select the one below that is the best fit for your research. If you are not sure, read the appropriate sections before making your selection.

☒ Life sciences ☐ Behavioural & social sciences ☐ Ecological, evolutionary & environmental sciences

For a reference copy of the document with all sections, see nature.com/documents/nr-reporting-summary-flat.pdf

Life sciences study design

All studies must disclose on these points even when the disclosure is negative.

Sample size	For all single cell analysis, the sample size was based on the number of single cells available in each dataset.
Data exclusions	No data has been excluded.
Replication	We used random seeds for model training to ensure that the models can be reproduced. We used 5 fold cross validation to compute classification scores for the applicable datasets. We used 5 fold cross validation with 20 repeats to compute regression scores for the applicable datasets. All attempts at replication were successful.
Randomization	We used random train/validation/test splits during training. We evaluated the best models based on performance on the validation dataset.
Blinding	Does not apply. The measurements in this dataset did not involve human subjects.

Reporting for specific materials, systems and methods

We require information from authors about some types of materials, experimental systems and methods used in many studies. Here, indicate whether each material, system or method listed is relevant to your study. If you are not sure if a list item applies to your research, read the appropriate section before selecting a response.

Materials & experimental systems

n/a	Involved in the study	
☒	☐	Antibodies
☐	☒	Eukaryotic cell lines
☒	☐	Palaeontology and archaeology
☒	☐	Animals and other organisms
☒	☐	Clinical data
☒	☐	Dual use research of concern
☒	☐	Plants

Methods

n/a	Involved in the study	
☒	☐	ChIP-seq
☒	☐	Flow cytometry
☒	☐	MRI-based neuroimaging

Eukaryotic cell lines

Policy information about cell lines and Sex and Gender in Research

Cell line source(s)	https://www.allencell.org/cell-catalog.html
Authentication	PCNA (AICS0088-83, https://hpscreg.eu/cell-line/UCSFi001-A-56)
Mycoplasma contamination	Stereility testing results can be found in the certificate of analysis for the cell line on https://www.allencell.org/cell-catalog.html. All cell lines tested negative for mycoplasma contamination.
Commonly misidentified lines (See ICLAC register)	No misidentified cell lines were used in this study.

Plants

Seed stocks	*Report on the source of all seed stocks or other plant material used. If applicable, state the seed stock centre and catalogue number. If plant specimens were collected from the field, describe the collection location, date and sampling procedures.*
Novel plant genotypes	*Describe the methods by which all novel plant genotypes were produced. This includes those generated by transgenic approaches, gene editing, chemical/radiation-based mutagenesis and hybridization. For transgenic lines, describe the transformation method, the number of independent lines analyzed and the generation upon which experiments were performed. For gene-edited lines, describe the editor used, the endogenous sequence targeted for editing, the targeting guide RNA sequence (if applicable) and how the editor was applied.*
Authentication	*Describe any authentication procedures for each seed stock used or novel genotype generated. Describe any experiments used to assess the effect of a mutation and, where applicable, how potential secondary effects (e.g. second site T-DNA insertions, mosiacism, off-target gene editing) were examined.*
